# Towards a Safer Future: Enhancing Vaccine Development to Combat Animal Coronaviruses

**DOI:** 10.3390/vaccines12030330

**Published:** 2024-03-19

**Authors:** Fusheng Si, Ruisong Yu, Shijuan Dong, Bingqing Chen, Chunhua Li, Shuai Song

**Affiliations:** 1 Institute of Animal Science and Veterinary Medicine, Shanghai Key Laboratory of Agricultural Genetics and Breeding, Shanghai Academy of Agricultural Sciences, Shanghai 201106, China; mr.fusheng@163.com (F.S.); yursong@163.com (R.Y.); dsjuan@saas.sh.cn (S.D.); c13919966733@163.com (B.C.); 2 Institute of Animal Health, Guangdong Academy of Agricultural Sciences, Scientific Observation and Experiment Station of Veterinary Drugs and Diagnostic Techniques of Guangdong Province, Ministry of Agriculture of Rural Affairs, and Key Laboratory of Livestock Disease Prevention of Guangdong Province, Guangzhou 510640, China

**Keywords:** one health, animal coronaviruses, vaccine, platforms and strategies, targets and efficacy

## Abstract

Coronaviruses (CoVs) are a large class of positively stranded RNA viruses that pose a significant threat to public health, livestock farming, and wild animals. These viruses have the ability to cross species barriers and cause devastating epidemics. Animals are considered to be intermediate hosts for many coronaviruses, and many animal coronaviruses also have the potential for cross-species transmission to humans. Therefore, controlling the epidemic transmission of animal coronaviruses is of great importance to human health. Vaccination programs have proven to be effective in controlling coronaviruses infections, offering a cost-effective approach to reducing morbidity and mortality, so the re-emergence of lethal coronaviruses emphasizes the urgent need for the development of effective vaccines. In this regard, we explore the progress in animal coronavirus vaccine development, covering the latest taxonomy of the main animal coronaviruses, spillover events, diverse vaccine development platforms, potential main targets for animal coronavirus vaccine development, and primary challenges facing animal coronavirus vaccines. We emphasize the urgent need to create a “dual-effect” vaccine capable of eliciting both cellular and humoral immune responses. The goal is to highlight the contributions of veterinary scientists in this field and emphasize the importance of interdisciplinary collaboration between the veterinary and medical communities. By promoting communication and cooperation, we can enhance the development of novel and super vaccines to combat human and animal coronavirus infections in the future.

## 1. Introduction

**Figure 1:**
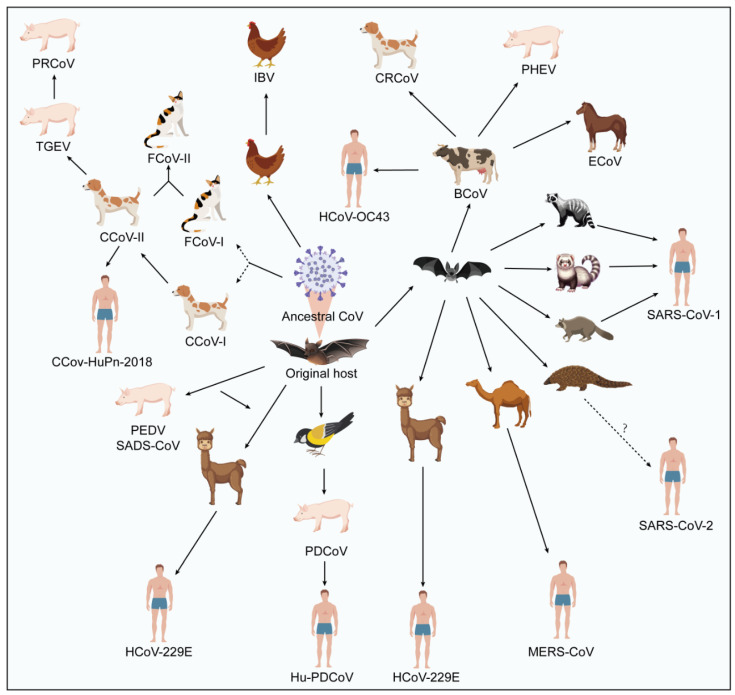
Schematic diagram showing zoonotic cycle of coronaviruses. Coronaviruses can infect various animal species, increasing the likelihood of cross-species transmission. Bats serve as natural reservoirs for ancestral coronaviruses. Direct transmission between host species, whether confirmed or suspected, is depicted by solid arrows, acknowledging the potential for indirect transmission through an unidentified intermediate host. Additionally, dashed arrows signify suspected indirect transmission via an unidentified intermediate host, with the understanding that direct transmission cannot be ruled out. Uncertain spillover events are represented by dotted arrows accompanied by a question mark.

CoVs are established pathogens that have been identified as the causative agents of respiratory and gastrointestinal illnesses in both animals and humans. Human coronaviruses (HCoVs) have been acknowledged as significant contributors to respiratory tract infections globally since their discovery in the 1960s. To date, there are nine known CoVs that infect humans, namely human coronavirus 229E (HCoV-229E), human coronavirus OC43 (HCoV-OC43), human coronavirus NL63 (HCoV-NL63), human coronavirus HKU1 (HCoV-HKU1), severe acute respiratory syndrome coronavirus (SARS-CoV), Middle East respiratory syndrome coronavirus (MERS-CoV), canine coronavirus-human pneumonia-2018 (CCoV-HuPn-2018), human porcine delta coronavirus (Hu-PDCoV), and the newly identified severe acute respiratory syndrome coronavirus 2 (SARS-CoV-2) (also known as 2019-nCoV) [1,2,3,4]. Apart from the human coronavirus, there are also animal coronaviruses. Animal coronaviruses have been identified in a wide range of domestic and wild animal species, including birds, pigs, cattle, dogs, cats, rodents, and bats (Figure 1). It is known that not only human coronaviruses pose a threat to public health, but animal coronaviruses also continue to emerge and cause diseases [5,6,7,8]. Certain coronaviruses found in animals have the potential to induce significant morbidity and mortality in their respective hosts, exemplified by pathogens like the porcine epidemic diarrhea virus (PEDV) and the feline infectious peritonitis virus (FIPV). Furthermore, there exists compelling evidence suggesting zoonotic transmission of several human coronaviruses, with animal reservoirs serving as their likely origins. Notably, the SARS-CoV outbreak of 2002–2003 was traced back to bats in China [9,10,11], while dromedary camels in the Middle East were responsible for the MERS-CoV outbreak in 2012 [12]. Although several plausible candidates have been proposed, there is no clear evidence for the involvement of specific animal intermediate hosts in the origin of SARS-CoV-2 [13,14,15]. In addition, zoonotic transmission has been documented for HCoV-OC43 (from cattle to humans) and potentially for HCoV-229E, with a suggested transmission route from bats to humans through camels [16,17,18]. The transmission of zoonotic viruses between humans and animals results in the development of severe respiratory diseases, including acute respiratory distress syndrome (ARDS) and pneumonia, often resulting in fatalities [19]. Hence, the inter-species transmission of animal coronaviruses to humans represents a critical concern, as it can precipitate the emergence of novel viral strains with pandemic capabilities (Figure 1).

It is widely recognized that traditional vaccination is the most effective approach for preventing and controlling CoV infections and transmissions. This is because vaccinations are more cost-effective than treatments and can substantially decrease morbidity and mortality rates in vaccinated populations. Given the zoonotic nature of animal coronaviruses, it is crucial to develop effective vaccines to control their spread and prevent future outbreaks. Although several animal coronavirus vaccines are currently available, their efficacy and ability to confer cross-protection against various strains and subtypes are limited [20,21]. Therefore, there is an urgent need to develop new generation vaccines that can provide broad protection against diverse animal coronaviruses. Thus, this review systematically summarizes the current status of animal coronaviruses vaccine and highlights future development directions. The concepts presented in this review are aimed at shedding light on vaccine development and viral prevention strategies for animal coronaviruses.

## 2. Main Animal Coronaviruses and Their Taxonomic Perspectives

**Figure 2:**
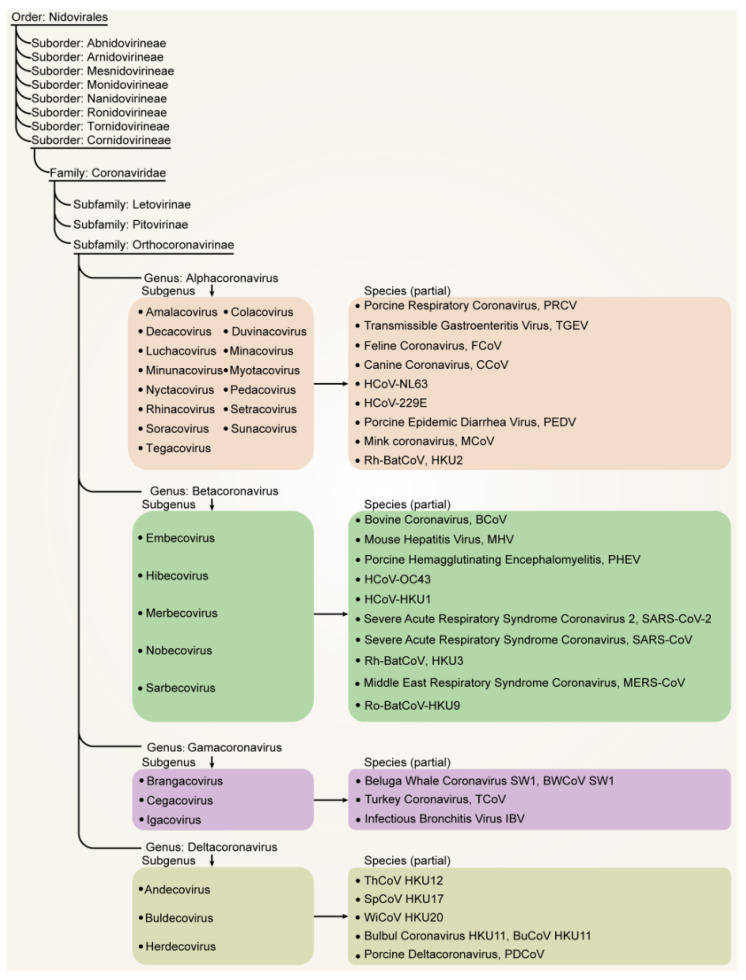
The latest classification of human and animal coronaviruses according to the International Committee on Taxonomy of Viruses (ICTV) [3,22,27]. The figure illustrates the classification of coronaviruses within the order *Nidovirales*, suborder *Cornidovirineae*, family *Coronaviridae*, and subfamily *Orthocoronavirinae*, which further divides into the alpha, beta, gamma, and delta genera. Partial species of each genus are depicted in the figure.

Based on the classification standards set by the International Committee on Taxonomy of Viruses (ICTV), the taxonomy of CoVs has been recently categorized within the order *Nidovirales* and the family *Coronaviridae*. This family has been subdivided into three distinct subfamilies: *Orthocoronavirinae*, *Letovirinae*, and *Pitovirinae* [22]. Within the *Orthocoronavirinae* subfamily, four genera have been identified based on genetic and serologic characteristics: *Alphacoronavirus (αCoV)*, *Betacoronavirus (βCoV)*, *Gammacoronavirus (γCoV)*, and *Deltacoronavirus (δCoV)* (Table 1, Figure 2) [3,23,24]. The latest ICTV classification reveals that the genus *αCoV* comprises 15 subgenera with 26 viral species, while the *βCoV* genus includes 5 subgenera and 14 viral species. *δCoV* and *γCoV* each consist of three subgenera, hosting seven and five species, respectively (Figure 2) [22,24]. In terms of host range, each genus of coronavirus demonstrates the ability to infect a diverse range of host species. Specifically, *αCoV* and *βCoV* predominantly infect mammals, particularly bats. On the other hand, *δCoV* and *γCoV* primarily infect birds, though they can also infect mammals [5]. The *Letovirinae* and *Pitovirinae* subfamilies are associated with amphibians and bony fish hosts, respectively [25]. Up to now, coronaviruses and coronavirus-like infections have been reported in various animal species, including swine, cattle, horses, camels, cats, dogs, rodents, birds, bats, rabbits, ferrets, mink, and wildlife, with many being subclinical [26]. It is believed that many animal coronaviruses can be transmitted across species to humans, posing a significant threat to public health. Below are some of the major animal coronaviruses that have been identified.

### 2.1. Porcine Coronaviruses

Porcine coronaviruses are a group of viruses that can cause severe diseases in pigs, leading to significant economic losses in the swine industry. They can be categorized into two types based on clinical signs: enteric and respiratory types. These types lead to fecal-oral transmission and aerogenic transmission as the main routes for viral spread, respectively. Recently, there are at least six known pig coronaviruses: PEDV, TGEV, SADS-CoV, porcine respiratory coronavirus (PRCV), porcine deltacoronavirus (PDCoV), and porcine hemagglutinating encephalomyelitis virus (PHEV) [62,63,64,65]. Several coronaviruses affect pigs. Among these, PEDV, TGEV, PRCV, and SADS-CoV belong to *αCoV*, while PHEV and PDCoV belong to the *βCoV* and *δCoV* genra, respectively [62]. Swine enteric coronavirus diseases are caused by PEDV, TGEV, SADS-CoV, and PDCoV. Compared to infections of piglets with SADS-CoV or PDCoV, PEDV and TGEV are the most important enteric coronaviruses in pigs, causing significant economic losses to the pig-farming industry worldwide. These viruses cause severe gastrointestinal diseases in neonatal pigs, affecting the respiratory and gastrointestinal tracts (TGEV, PEDV, PDCoV, and SADS-CoV), as well as the peripheral and central nervous systems (PHEV). Although natural infection stimulates protective immunity, cross-protection among these viruses is not yet known [62].

Here, our focus is on introducing three coronaviruses that have been extensively studied in pigs. PEDV was first identified in the 1970s in the United Kingdom, and has since been documented in various countries globally. However, the virus was not detected in the United States until 2013, causing a widespread outbreak [66]. Similar to TGEV, the virus triggers severe diarrhea in piglets, leading to significant economic losses. PEDV antibodies are incapable of neutralizing TGEV, suggesting antigenic variations between the two viruses. PEDV exhibits some genetic characteristics that resemble human coronaviruses, particularly HCoV-229E. Moreover, similar to SARS-CoV and SARS-CoV-2, PEDV is capable of replicating in Vero cells [67,68,69,70,71], indicating potential similarities in the pathogenicity mechanisms between the coronaviruses. TGEV was first identified in 1946 [72]. In recent years, several vaccines have been tested for their efficacy in safeguarding against TGEV [73,74,75]. Yet, it has been observed that administering a live-attenuated TGEV vaccine to pregnant pigs led to elevated antibody levels in their serum and colostrum. Despite this, the antibody levels in the milk dropped significantly within days after giving birth, indicating that the live-attenuated TGEV vaccine does not offer sufficient protection for their nursing offspring [38,76]. Additionally, direct inoculation of young pigs with attenuated virus is also incapable of inducing enough immunoglobulin A (IgA)-secreting cells in the intestines to offer protection against TGEV. Interestingly, sows that have recovered from a virulent TGEV infection produce enough milk IgA to provide protection against infection and diarrhea in their suckling offspring [76]. PHEV is currently the sole recognized neurotropic coronavirus impacting swine and is the only identified porcine *βCoV* to date. The initial report of PHEV dates back to approximately 1957 in Ontario, Canada, and subsequent occurrences of outbreaks have been well-documented [77,78]. Despite its ubiquitous presence in most swine herds globally, PHEV infection often does not cause clinical signs. The disease caused by PHEV is age-dependent, with morbidity and mortality affecting piglets under four weeks of age. While pigs are the only known species susceptible to natural PHEV infection, experimental infections of mice and Wistar rats show that the virus is also neurotropic. Currently, no vaccine has been developed to protect against PHEV.

From an epidemiological perspective, TGEV, PRCV, and PHEV have been present in the pig population for several decades, while PEDV, PDCoV, and SADS-CoV are considered emerging coronaviruses [62]. Despite infecting the same natural host, these six CoVs employ different cellular receptors for binding, and usually cause infections in pigs. However, PDCoV has demonstrated the ability to infect other species, such as badgers, calves, and cats [79], and even has the potential to infect humans [2,80]. Of particular concern is PEDV, which has been found to engage with human APN and replicate in human intestinal cells, indicating the potential for cross-species transmission [40,81]. Therefore, there is a need to strengthen coronavirus surveillance in other possible reservoirs, as both PDCoV and PEDV may pose a potential risk to other animals and humans. The emergence of novel pig coronaviruses and their potential to cause zoonotic infections highlights the importance of understanding the epidemiology and public health implications of these viruses [64,82].

### 2.2. Canine Coronavirus (CCoV)

Canine CoVs, which comprise canine coronavirus (CCoV) and canine respiratory coronavirus (CRCoV), belong to the *αCoV* and *βCoV* genera, respectively. Currently, two genotypes of CCoV are acknowledged, identified as CCoV types I (CCoV-I) and II (CCoV-II) [83]. These genotypes differ primarily in their spike proteins, which share only around 50% similarity [84]. CCoV type I and type II have evolutionary links to feline CoV (FCoV) type I and type II, respectively. FCoV type II arose from a heterologous recombination between FCoV type I and CCoV type II, while CCoV type I shares greater genetic similarity with FCoV type I than with CCoV type II [85]. The first case of canine CoV was reported in Germany in 1971 [86], and since then, multiple CCoV outbreaks have been documented worldwide, underlining the significance of CCoV as an enteropathogen of dogs [83]. Canine CoV enters enterocytes lining the small intestine villi via the host protein aminopeptidase (APN). Noteworthy is the fact that while CCoV genotype II engages canine APN, it also interacts with feline APN, contrary to the conventional idea that each virus must utilize a species-specific receptor [28].

CCoV, characterized by its high infectivity, predominantly spreads through fecal shedding, and transmission occurs primarily through the fecal-oral route. This virus exhibits a specific tropism for the alimentary tract, leading to distinct clinical manifestations, typified by gastroenteritis symptoms, including anorexia, emesis, watery diarrhea, and dehydration in canines. Despite its high morbidity rates, CCoV has low mortality rates [84]. Recent studies have shown that CCoV strains have the capability of experiencing S gene exchange with TGEV at the N-terminal domain, providing a new and unexplored factor in the evolution process of CCoV and the potential inter-species circulation between dogs and pigs [87]. In contrast, CRCoV induces a mild respiratory illness in canines, characterized by clinical symptoms such as coughing and potential bronchopneumonia. Remarkably, CRCoV exhibits notable genetic and amino acid resemblances to bovine CoV (BCoV), suggesting a plausible evolutionary link from a shared ancestor [88]. The mechanism of CRCoV binding and entry involves sialic acids, alongside the potential participation of human leukocyte antigen class I (HLA1) [29,89]. Both inactivated and attenuated vaccines demonstrate efficacy in preventing canine CoV infection.

### 2.3. Equine Coronavirus (ECoV)

The equine coronavirus (ECoV), classified within the *βCoV* genus, was first discovered in the feces of a diarrheic foal in North Carolina, USA in 1999 (ECoV-NC99) [90]. Since 2010, various outbreaks have been reported in Japan, Europe, and the USA. ECoV is transmitted via the fecal-oral route, and horses contract the virus by consuming fecally contaminated feed and water. Infected horses, whether clinically symptomatic or asymptomatic, seem to be responsible for direct and indirect transmission of ECoV. Clinically, ECoV infection is associated with fever, lethargy, anorexia, colic, and diarrhea [91]. The disease is usually self-limiting, and horses typically recover with supportive care.

### 2.4. Feline coronavirus (FCoV) and Feline Infectious Peritonitis Virus (FIPV)

FCoV is a relatively harmless enteric or chronic asymptomatic infection, commonly found in domestic cats. The virus was identified as a coronavirus by electron microscopy in 1970 [92]. Biologically, FCoVs can be divided into two subtypes, namely the feline enteric virus CoV (FECV) and the feline infectious peritonitis virus (FIPV). APN serves as a binding receptor for both FECV and FIPV [93]. FECV typically causes inapparent enteritis in cats by replicating in the intestinal epithelium [94]. On the other hand, FIPV infects monocytes and can lead to systemic diseases, including fatal peritonitis with immune complex vasculitis, accompanied by necrosis and pyogenic granulomatous inflammation [95].

FCoVs can be categorized into two serotypes, I and II, based on differential antibody neutralization and variations in the amino acid sequence of the S protein [96]. Serotype I FCoV predominates as the most prevalent strain, and recombination events between serotype I FCoV and serotype II canine CoV can directly yield serotype II FCoV (Figure 1). From a clinical perspective, it is challenging to distinguish between FECV and FIPV based on virus antigen, virus particle morphology, and serology [97]. Due to the potential occurrence of antibody-dependent enhancement (ADE) following FIP vaccination [98], vaccination against FIP is currently not recommended. Further research is necessary to explore the pathogenesis of FIPV infection and in order to develop novel vaccines that are highly efficient and safe.

### 2.5. Bovine CoV

In 1973, bovine CoV (BCoV) was initially identified as a cause of calf diarrhea in the United States [99]. This virus belongs to the *βCoV* of the family *Coronaviridae* and is capable of causing neonatal diarrhea, winter dysentery, and respiratory illness in cattle [51,100]. BCoV attachment and entry into host cells relies upon the binding of sialic acids as receptors. It can be transmitted through fecal-oral or respiratory means and has a dual tropism for both the respiratory and gastrointestinal tracts [101,102,103]. It has been suggested that the HCoV-OC43 is related to BCoV and that BCoV is likely an ancestor of HCoV-OC43, or they may have evolved from a common ancestor [101].

In addition to infecting cattle, BCoV has been detected in other hosts, such as wild ruminants, dogs, poultry, giraffes, and it may have zoonotic potential [101,102,103]. BCoV, like other members of the Coronaviridae family, expresses a surface S glycoprotein, which carries a furin cleavage site and was cleaved into S1 and S2 subunits. However, unlike many other Beta coronaviruses, it possesses a hemagglutinin esterase (HE) that appears to have been acquired through recombination and resembles the hemagglutinin of influenza C virus. Both S and HE proteins aid in viral attachment to host cells and induce the formation of neutralizing antibodies against BCoV [51,100]. A common practice for controlling BCoV infection in cattle is the vaccination of pregnant cows, which can protect neonates through the transfer of antibodies via the colostrum [104].

### 2.6. Avian CoVs

The primary virus belonging to avian CoV is the infectious bronchitis virus (IBV) in chickens. It belongs to the *γCoV* genus and exhibits high serotype diversity owing to selection pressures, including natural selection, genetic evolution, and human intervention [105,106]. IBV infection can lead to severe respiratory, urogenital, renal, and reproductive disorders, manifested by symptoms such as rales, sneezing, diarrhea, and reduced egg quality and production [107,108]. The sialic acid acts as a receptor determinant for avian IBV entry into host cells [59,109].

Other avian species that have been confirmed to be susceptible to CoV-induced diseases include turkeys, pheasants, and guinea fowl [110]. Of these, Turkey coronavirus (TCoV) is the most thoroughly characterized and economically significant after IBV, having been identified as a cause of enteric disease in turkeys in the US since the 1940s and currently posing a problem in turkey-producing regions worldwide. Pheasant CoV (PhCoV) is implicated in respiratory and renal issues, and seems closely related to IBV and TCoV. Guinea fowl CoV (GfCoV) is linked to a fulminating disease, with a high death rate and possibly pancreatic degeneration [111]. Live-attenuated vaccines and inactivated oil-emulsion vaccines are commonly employed for the control of IBV infections in farms [112,113,114]. However, no vaccines are available for TCoV, PhCoV, or GfCoV.

## 3. Genomic Structure of Animal CoVs and Function of Their Related Proteins

**Figure 3:**
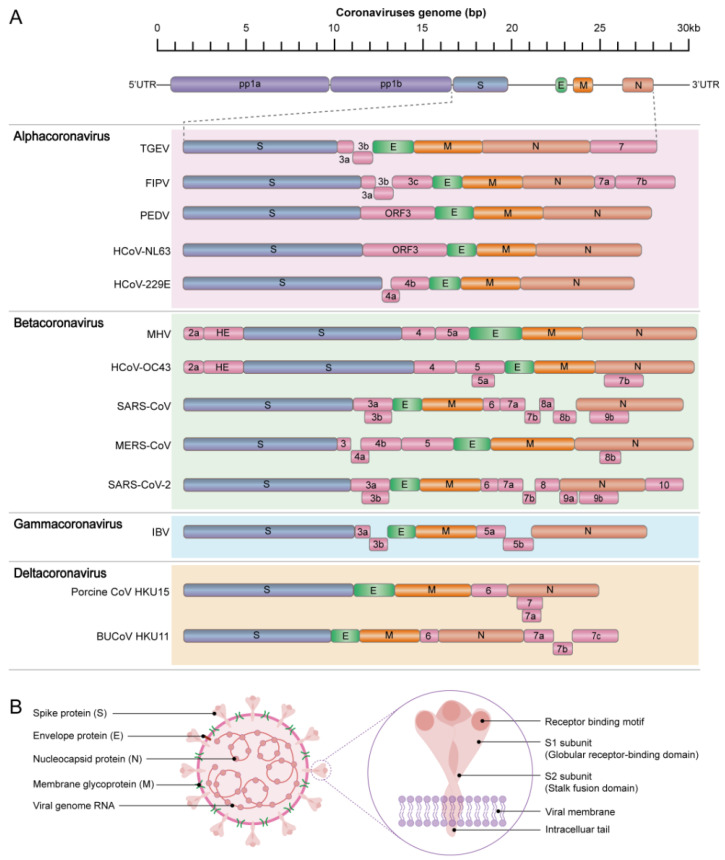
Schematic diagram of coronavirus structure and genome organization. (**A**) Illustration of the gene, protein, and genome organization of coronaviruses. Coronaviruses possess a positive-sense, single-stranded RNA (ssRNA) genome, ranging from 22 to 36 kb. The 5′-terminal portion encodes a polyprotein, pp1ab, cleaved into 16 non-structural proteins involved in genome transcription and replication. The 3′ terminus encodes structural proteins—spike (S), envelope (E), membrane (M), and nucleocapsid (N). Additionally, species-specific accessory genes, dispensable for virus replication, are present. A comparison is made between prototypical and representative strains across the four coronavirus genera. (**B**) Schematic representation of the coronavirus viral particle, highlighting the spike (S) protein and receptor-binding domain (RBD) as primary inducers of neutralizing antibodies. These components constitute essential antigens for coronavirus vaccines. Also depicted are the N (nucleocapsid), M (membrane), and S (spike) proteins, crucial targets for subunit vaccine development.

Much like their human counterparts, animal CoVs have a genome consisting of a linear, positive-sense, single-stranded RNA of approximately 22,000 to 36,000 nucleotides, with a 5′-cap structure and a 3′-polyadenylated tail [115]. They have a genome structure that comprises two open reading frames (ORFs) located in the 5′-proximal two-thirds of the genome [35,38,116]. These ORFs, known as ORF1a and ORF1b, encode the replicase polyproteins pp1a and pp1ab [117], as shown in Figure 3. The 3′-proximal one-third of the genome encodes four structural proteins: S, E, M, and N, along with several accessory proteins [38]. Some coronaviruses also have an additional structural protein called the hemagglutinin-esterase (HE) protein, which is acquired through recombination events (Figure 3A) [118].

The S protein serves a pivotal role in facilitating viral entry into host cells through interaction with key receptors, such as ACE2, APN, and DPP4 (Table 1). This protein comprises two distinct subunits: the N-terminal S1 subunit and the C-terminal S2 subunit (Figure 3B) [119,120]. The S1 subunit further encompasses an N-terminal domain (NTD) and a C-terminal domain (CTD), also known as the receptor-binding domain (RBD). While the NTD binds to attachment factors, the CTD (RBD) orchestrates virus-host cellular receptor binding (Figure 3B) [120,121]. Conversely, the S2 subunit facilitates the fusion between viral and host cell membranes [122]. Furthermore, various structural proteins, such as N, M, and E proteins, exhibit diverse functionalities throughout the viral life cycle and pathogenesis [123,124]. For example, the N protein is indispensable for processes such as RNA synthesis, replication, virion assembly, and post-translational modification [125,126], whereas the M protein interacts with the N protein and aids in virion assembly [127,128]. The E protein functions as a virulence determinant by creating ion channels within lipid bilayers, thereby contributing significantly to virion assembly, budding, and release [129,130]. Notably, certain coronaviruses, such as mouse hepatitis virus (MHV), HCoV-HKU1, HCoV-OC43, and BCoV, possess an additional structural protein, the hemagglutinin esterase (HE) protein, positioned upstream of the S protein gene (Figure 3A). Both the S and HE proteins play crucial roles in facilitating virus attachment to host cells [118,122].

In addition, coronaviruses also have accessory proteins encoded in the 3′ terminal region of the genome, which vary in number and type across different coronaviruses (Figure 3A). These proteins are generally considered non-essential for virus replication in vitro. However, they contribute to viral–host protein interaction and participate in many processes, including virus particle assembly, apoptosis, autophagy, and inflammatory response. These functions are crucial to viral pathogenesis [68,131,132,133,134,135].

## 4. Spillover Event and Cross-Species Potential of Animal Coronavirus

CoVs have been responsible for various diseases in numerous hosts, resulting in high mortality rates and significant economic losses. The diverse genomic propensity of CoVs facilitates their ability to evolve and recombine, enabling them to overcome natural barriers to cross-species transmission, allowing the virus to adapt and proliferate in new hosts during a spillover event [5]. For instance, the recent spillover of bat CoVs into pigs, which includes PEDV and SADS-CoV, is an example of such events. Moreover, documented cases of animal-to-human transmission via intermediate hosts, such as SARS-CoV, potentially SARS-CoV-2, and MERS-CoV, highlight the critical role of intermediate host species in facilitating viral spread from bats to humans. As CoV spillovers can occur undetected among animal populations, understanding and preventing this event occurrence are increasingly crucial in mitigating cross-species transmission.

Similarly, coronaviruses have been observed to emerge within the swine industry as a result of spillover events with bats. One such example is transmissible gastroenteritis (TGE), which was first identified as a CoV (TGEV) in swine in the United States in 1946 [63,72,136]. Interestingly, PRCV evolved from TGEV into a respiratory pathogen in 1984, indicating the capacity of CoV mutation to confer varying tissue tropism within a single host species. Moreover, PEDV is suspected to have originated from a bat reservoir [137]. PDCoV is also thought to have emerged from a bird-to-pig transmission event, although its specifics remain unknown [5]. Ongoing incidents of coronavirus spillover are occurring in pigs, with one example being SADS-CoV. This *βCoV* strain appears to have directly jumped from bats to pigs in China [138,139,140]. The existence of spillover, spillback, and secondary spillover has been confirmed in SARS-CoV-2 [141], demonstrating the wide range of hosts and ongoing transmission events observed in domestic, captive, and wild animals. This underscores the urgent need for further research into animal coronaviruses. Thus, preventing the spread of the coronavirus in intermediate hosts, along with the development of new vaccines targeting the virus, is an effective measure to control the spread of animal coronavirus. Indeed, the “One Health” concept emphasizes the need for close monitoring of the health status of animals and humans in certain environments, accelerating the development of animal vaccines to mitigate the possibility of further disease outbreaks. These efforts aim to minimize the spread of animal infectious diseases and enhance the capacity to prevent the transmission of future emerging infectious diseases.

## 5. Types of Vaccine Development Platforms and the Trialed or Generated Animal Coronavirus Vaccines

**Figure 4:**
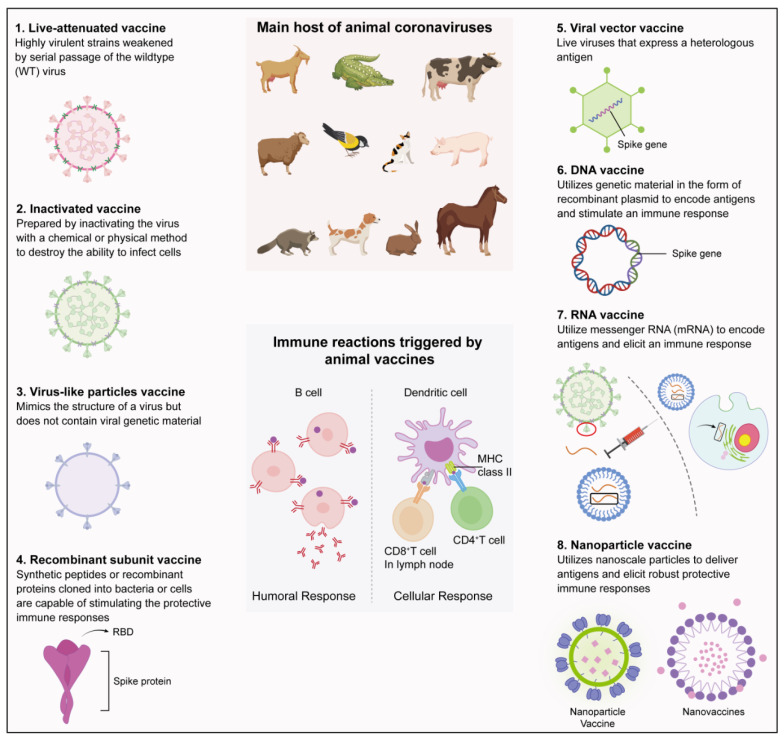
Vaccine development platforms of animal coronavirus. The left and right panels provide a schematic overview of various coronavirus vaccine platforms, encompassing the live-attenuated vaccine, inactivated vaccine, virus-like particles vaccine, recombinant subunit vaccine, viral vector vaccine, DNA vaccine, RNA vaccine, and nanoparticle vaccine. The upper portion of the middle panel represents the primary hosts of animal coronaviruses, while the lower portion depicts the two main immune responses elicited by animal coronavirus vaccines in vivo: cellular and humoral immune responses.

Viral vaccines are primarily intended to trigger the immune system of the body, which is mainly accomplished by activating B cells that produce antibodies and generating killer T cells, also known as cytotoxic T cells. This allows the immune system to recognize and respond swiftly to specific viral pathogens in the future. Several significant vaccine development platforms (including classical vaccine platforms and next-generation vaccine platforms) and their working mechanisms are outlined below, some of which have not yet been employed in the development of vaccines against animal coronaviruses (Figure 4) (Table 2).

Classical vaccine platforms, such as live attenuated, inactivated, viral vector, subunit, and virus-like particles (VLPs) vaccines, are widely used [167]. Live-attenuated vaccines are derived from virulent virus strains and weakened through passages on host animals or cells, or by genetic modifications. Inactivated vaccines are viral particles rendered inactive through physical or chemical methods. Viral vector vaccines use modified viruses to deliver antigens into cells. Adenovirus, retroviruses, and vaccinia viruses are the primary viral vectors traditionally employed for this purpose [168]. Moreover, some animal viruses, including porcine reproductive and respiratory syndrome virus (PRRSV), swine pox virus (SPV), recombinant Newcastle disease virus (rNDV), and recombinant chimeric TGEV-PEDV virus have also proven effective in expressing foreign genes from animal viruses [169,170]. Additionally, genome editing methodologies, including CRISPR-Cas9 and reverse genetic strategies, are extensively utilized in the development of viral vector vaccines [171,172]. Subunit vaccines contain the essential immunogenic components of a pathogen. VLPs mimic the virus structure without its genetic material, self-assembling with viral proteins to resemble the native virus.

Apart from the classical vaccine platforms, the next-generation vaccine platforms, such as nanoparticle vaccines and nucleic acid vaccines, are also wildly used in coronavirus vaccine development. Nanoparticle vaccines are a novel type of vaccine that utilizes the unique properties of nanoparticles to deliver antigenic proteins to immune cells in the body. These vaccines consist of small particles, usually between 1–100 nm in size, which are engineered to carry the antigenic proteins of the pathogen [173]. In addition, nucleic acid vaccines, including DNA and mRNA vaccines, utilize genetic material from a pathogenic microorganism, coupled with gene injection technology, to elicit an immune response against it. DNA vaccines are generated through the insertion of a gene that encodes a specific or multivalent antigen into a bacteria-derived recombinant plasmid. This plasmid must be controlled by a powerful promoter to elicit both cellular and humoral responses [174]. An alternative to the DNA vaccine is the mRNA vaccine, which utilizes a copy of the messenger RNA molecule to elicit an immune response [175,176]. These mRNA vaccines come in two different forms: non-amplifying mRNA (conventional mRNA) and self-amplifying mRNA. These two types differ in their mechanisms of action [177]. By harnessing the host cell machinery, mRNA vaccines facilitate in vivo translation of mRNA into antigens, generating robust humoral and cellular immune responses that resemble those of viral infections. For example, an mRNA lipid nanoparticle (mRNA-LNP) vaccine created by Li’s group, which contains the complete PEDV spike (S) protein, demonstrated the ability to stimulate strong PEDV-specific immune responses in piglets. This vaccine not only effectively shielded actively immunized piglets from PEDV infection but also imparted passive anti-PEDV immunity to neonatal piglets through the transfer of colostrum-derived antibodies from immunized sows [157]. mRNA vaccines have shown great efficacy in fighting novel coronavirus infections in humans. With their ability to stimulate robust immune responses for both active and passive immunity, the design strategies and application of these vaccines are now being integrated into the creation of vaccines for animal coronaviruses.

In addition to the vaccine development platforms mentioned above, there is a new type of vaccine development platform—plant-based vaccine expression platform. Currently, this expression strategy is increasingly being applied in the development of animal vaccines. For example, Xu et al. used rice to produce a super vaccine for swine fever [178]. This study demonstrated the potential of the rice expression system to precisely express designed proteins in vitro by expressing the “human-shaped” E2 dimer (ht-rE2 dimer) in rice. The expressed E2 dimer exhibits the natural conformation of viral envelope proteins and high antigenic activity, while also being safe, easy to produce on a large scale, and cost-effective. This research provides a theoretical basis for using the rice expression system to produce animal vaccines. Additionally, the same team proposed a universal “head-to-tail” dimeric vaccine antigen model and successfully prepared a highly efficient recombinant antigen, Osr2HN, using the rice endosperm expression system [179]. This antigen displays multiple epitopes with appropriate distances, which can effectively activate B cells. Further animal challenge tests have shown that this antigen design significantly enhances the immune response of subunit vaccines, leading to a more efficient antibody-generation effect [179]. The effective utilization of the plant-based vaccine development platform in creating swine fever and paramyxovirus vaccines underscores the importance of expediting the investigation and utilization of this platform for the development of animal coronavirus vaccines. This will aid in the timely development of a highly effective animal coronavirus vaccine.

## 6. Status of Vaccine Development for Animal Coronaviruses

Ongoing efforts to develop effective vaccines against animal coronaviruses have made significant progress in recent years. Notably, vaccines for porcine coronaviruses (e.g., TGEV and PEDV) and avian coronaviruses (e.g., IBV) have demonstrated the highest level of technical maturity and rapid advancement. TGEV and PEDV, which cause diarrhea in pigs, have been addressed through the development and extensive use of inactivated or attenuated virus-based vaccines that effectively prevent and control infections [180]. While vaccines for TGEV have long been available, those for highly virulent PEDV strains continue to pose a challenge [65]. For example, G1a PEDV-based vaccines, including both inactivated and live-attenuated forms, were effectively utilized to manage PEDV outbreaks in Asia before 2010. A previous study demonstrated the development of an inactivated vaccine derived from a cell-adapted CV777 strain, which yielded high protection rates in piglets through the passive immunization of vaccinated sows [181]. Subsequently, a bivalent live-attenuated vaccine for PEDV was successfully created, providing passive protection rates against the virus in China [182]. Significantly, Japan and South Korea have also developed vaccines, with South Korea producing two attenuated virulent strains, SM98-1 and DR13 [148,149]. Additionally, the Japanese strain 83P-5, attenuated through serial passages in Vero cells, is commercially available as a live-attenuated vaccine [147]. These bivalent vaccines effectively curbed the spread of PEDV in China until the emergence of highly virulent PEDV variants at the end of 2010. Since late 2010, China faced severe PED outbreaks [183], necessitating vaccines targeting G2 strains. In 2015, China officially approved and introduced two multivalent vaccines [184]. One is a trivalent vaccine derived from attenuated strains of PEDV (the CV777 strain), TEGV, and porcine rotavirus. The other is a bivalent attenuated vaccine comprising strains of TGEV and PEDV (the ZJ08 strain, G1b). However, their effectiveness is debatable due to inadequate cross-protection against G1 and G2 strains [185]. Given this situation, other candidate PEDV vaccine strains are also under intense development. It has been reported that the deactivation of a potential PEDV vaccine can stimulate a strong immune response and offer protection [186,187]. Additionally, it has been demonstrated that deactivating both the 2′-O-MTase and the endocytosis signal of the spike protein can be an effective approach in designing a promising live-attenuated vaccine for PEDV [188]. Furthermore, the safety and effectiveness of two attenuated PEDV vaccine candidates, specifically the emerging non-S INDEL PEDV strain PC22A at the 100th cell culture passage level and at the 120th passage level (P120), were previously evaluated and showed promising effects in weaned pigs [189]. Effective and safe vaccines for virulent PEDV strains still remain unavailable [190], as classical PEDV vaccines have failed to combat these strains in Asia [191]. Presently, new-generation vaccine development platforms have been established, including the PEDV vaccine platform utilizing a bacterial artificial chromosome (BAC) and a genome recombination-resistant platform facilitated by the RMT mutant [192,193]. However, further observation is necessary to evaluate its efficacy in PEDV vaccine development.

With the emergence of other porcine coronaviruses, such as PDCoV and SADS-CoV, the development of new vaccines against these viruses is crucial [38]. Therefore, recent studies have focused on the development of new vaccines against PDCoV and SADS-CoV. A study reported the development of a live-attenuated vaccine against SADS-CoV that was shown to be safe and effective in protecting pigs against SADS-CoV infection [38]. Similarly, a study reported the development of a recombinant PDCoV spike protein vaccine that was shown to be effective in inducing neutralizing antibodies against PDCoV [194]. These findings suggest that the development of new vaccines against pig coronaviruses is feasible and can help to prevent and control the spread of these viruses.

The IBV vaccine stands as another example of animal coronavirus vaccines with well-established technology and notable effectiveness. The vaccination has been shown to be a safe and effective measure in protecting chickens against IBV, and the development and ongoing assessment of live-attenuated vaccines further highlight the extensive and ongoing efforts to optimize vaccine development in this field. Specifically, the utilization of the H strain of avian infectious bronchitis virus represents a time-honored approach in IBV vaccine development [195]. Furthermore, a live-attenuated vaccine targeting IBV has been formulated and is presently undergoing rigorous clinical trials [196,197,198].

For feline coronavirus, the first vaccine for FIP was licensed in 1991 [199]. Presently, a commercially available vaccine for this purpose exists in various countries [200,201,202]. Previous research has indicated that vaccination against feline coronavirus infections in cats presents both advantages and disadvantages, as a modified live FIP vaccine was demonstrated to be safe and effective under field conditions [143]. However, certain kittens who were infected with naturally occurring feline coronavirus exhibited adverse reactions to the vaccine, with three cats developing FIP within the first month after vaccination [203]. These findings underscore the need for the development of new vaccines targeting epidemic/variant strains. Researchers are currently exploring the feasibility of developing a vaccine for feline coronavirus utilizing a small molecule, XM-01, as an inactivated vaccine [204]. Other veterinary vaccines designed to combat animal coronaviruses are commercially obtainable in the European countries, including vaccines developed to prevent shipping fever in young calves from bovine coronavirus infection and to curb canine enteric coronavirus infections in dogs [201,205]. Despite these advancements, vaccines currently available on the market have demonstrated limited efficacy against canine coronavirus [206].

## 7. Main Targets for Animal Coronavirus Vaccine Development

**Figure 5:**
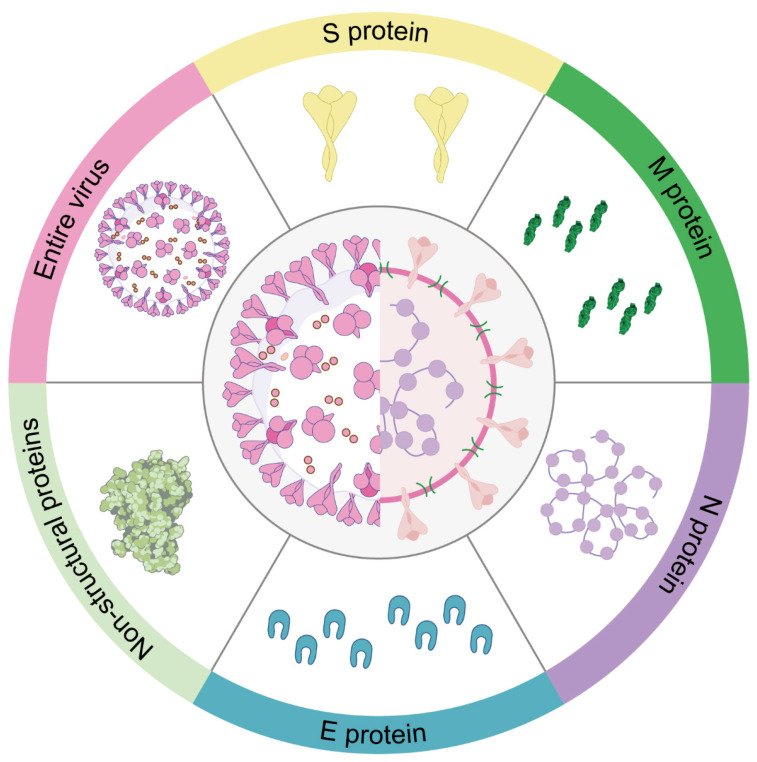
The figure shows the six targets being used as vaccines for animal coronavirus.

Understanding the role of different target proteins in the coronavirus life cycle is an important area of ongoing research for the development of effective vaccines against coronaviruses. The following figure highlights several target proteins used for the development of coronavirus vaccine (Figure 5).

### 7.1. S protein

Due to its ability to induce neutralizing antibodies against the pathogen, the spike (S) protein is a crucial target for most coronavirus vaccines, including those developed for COVID-19. The S protein consists of two subunits. The S1 subunit recognizes the receptor through its receptor-binding domain (RBD), while the S2 subunit facilitates the fusion of the virus with the membrane, enabling entry into the cell [120]. The S protein plays a pivotal role in the virus’s endocytosis by binding to the corresponding viral receptor, making it a prime target for the institution of the subunit vaccine [207]. The S1 subunit, encompassing both the RBD and the NTD, plays a pivotal role in binding to host receptors and serves as a prominent target in vaccine design [207]. Vaccination with RBD elicits specific antibodies that hinder receptor recognition, thus effectively blocking viral entry [120]. Notably, a majority of coronavirus subunit vaccines in development target the RBD. Moreover, the NTD of S proteins from diverse animal coronaviruses have demonstrated carbohydrate receptor-binding activity, as observed in TGEV and IBV [208,209]. Recent studies have also indicated further mutations in the antigenic site S1° and COE of the PEDV S protein, making it a main candidate target for animal vaccine development [209].

### 7.2. N Protein

Coronavirus contains a highly conserved N protein, which is the most abundant protein in the virus, with a molecular weight of approximately 50 kDa. This protein serves multiple functions, including the formation of nucleocapsids, virus budding, RNA replication, and mRNA transcription [210]. Although vaccine development has primarily focused on the S protein, the N protein has also been studied due to its major target for antibody responses and T-cell epitopes [211]. Research has demonstrated that vaccines targeting the N protein can induce robust T-cell responses and offer protection against coronavirus infections [125,212]. For instance, in vaccinated C57BL/6 mice, a DNA vaccine encoding the SARS-CoV N protein stimulated potent N-specific humoral and cellular immune responses, effectively reducing the viral titer of the challenging vaccinia virus [213]. Notably, studies on the avian infectious bronchitis virus revealed that the N protein is associated with the induction of cytotoxic T lymphocytes (CTLs), which correlated with decreased clinical signs and viral clearance from the lungs [214,215]. This suggests the crucial role of cellular responses in N protein-mediated protection. Furthermore, N-specific antibodies have been observed to confer protection against mouse hepatitis virus by engaging Fc-mediated effector functions [216]. However, it is worth noting that the N protein is generally less immunogenic compared to the S protein, and vaccines targeting the N protein may not be as effective as those targeting the S protein [125,212]. The issue of balancing viral clearance and immunopathogenesis complicates the development of N protein-based vaccines for COVID-19. As a result, no N protein-based vaccine has been reported thus far.

### 7.3. M Protein

The M protein is a structural protein that resides in the envelope of coronaviruses and is responsible for shaping the viral particle, as well as for virus assembly and release. During viral entry, the M protein plays a critical role in the interaction between the viral envelope and the host cell membrane. Recent research has highlighted the potential of the M protein as a target for the development of animal coronavirus vaccines, owing to its high level of conservation across different coronaviruses [217]. Immunogenic and structural analyses have revealed a T-cell epitope cluster within the transmembrane domain of the M protein, capable of eliciting a robust cellular immune response [218]. Moreover, studies have reported the efficient induction of neutralizing antibodies in SARS patients upon immunization with the full-length M protein [219]. Collectively, these findings underscore the potential candidacy of the M protein as a target for the development of vaccines against animal coronaviruses. Nonetheless, further research is needed to determine the most effective vaccine formulation and delivery strategy for M protein-based vaccines, as well as to evaluate their efficacy and safety in various animal species.

### 7.4. E Protein

In comparison to the spike (S), nucleocapsid (N), and membrane (M) proteins, the envelope (E) protein is not suitable as an immunogen due to its small ectodomains for immune cell recognition and small molecular sizes [220]. One reason for this inadequacy is that E proteins in different coronaviruses possess channel activity, which restricts their immunogenicity. Additionally, the E protein is a small membrane protein that is considerably less effective in generating an immune response compared to the S protein [221,222]. Experimental studies have demonstrated that sera from vaccinated donors, which received a vaccine employing a virus vector expressing the E protein, did not provide protection against SARS-CoV-2 infection [220]. Nevertheless, a more recent study demonstrated that SARS-CoV-2 E-protein had a stronger connection with the MHCs and lower solvent accessibility, which suggests the potential of the E protein as a target for SARS-CoV-2 vaccine development [223]. Consequently, further research is required to establish whether the E protein is a feasible target for the development of coronavirus vaccines.

### 7.5. Non-Structural Proteins (NSPs)

The non-structural proteins (NSPs) are emerging as promising targets for the development of coronavirus vaccines, as they are intricately involved in the virus’s replication and evasion of the host immune response. Despite being less studied than the structural proteins, the NSPs play a critical role in the virus’s behavior and pathogenesis [69]. Among the various NSPs, the papain-like protease (PLpro), the RNA-dependent RNA polymerase (RdRp), and the 3-chymotrypsin-like protease (3CLpro) have been identified as potential targets for vaccine development [224,225,226]. The RdRp, an enzyme responsible for replicating the virus’s RNA genome, holds promise as a target for both antiviral drugs and vaccines [227,228,229]. Similarly, the PLpro and 3CLpro are proteases that cleave viral polyproteins into functional NSPs, making them attractive targets for vaccine development [230,231]. Inhibiting these proteases could potentially prevent the virus from replicating and aid in therapeutic intervention. Currently, numerous vaccine candidates targeting NSPs are undergoing preliminary trials, necessitating further research to determine their efficacy [232,233].

### 7.6. The Entire Virus as a Target

The entire virus vaccines, including inactivated virus and live-attenuated virus vaccines, use the whole virus as vaccine targets. Along with structural proteins, such as the spike (S), nucleocapsid (N), and membrane (M) proteins [220,222,234,235] and non-structural and accessory proteins, they are another target for animal coronavirus vaccine development. They can provide a broader range of viral antigens for the immune system to recognize, which could lead to more effective protection against different strains of the virus [220]. Animal vaccines containing live-attenuated viruses have received licensing for IBV, TGEV, BCoV, and FIPV [211]. Nevertheless, researchers have shown that immunity tends to decrease over time following IBV and BCoV vaccines [211], raising concerns about the longevity of the immune response generated by vaccines that use the entire virus. Additionally, developing whole virus vaccines requires careful consideration of safety concerns, such as ensuring that the virus is sufficiently attenuated or inactivated to prevent disease, while still eliciting an effective immune response [235]. Hence, although entire virus vaccines hold promise for the development of coronavirus vaccines for animals, additional investigation and evaluation are necessary to ensure their safety and effectiveness.

## 8. Challenges in the Development of Vaccines against Animal Coronavirus

In light of the COVID-19 pandemic, significant advancements have been made in the realm of coronavirus vaccines. However, it is essential to acknowledge the presence of novel challenges in the domain of animal coronavirus vaccine research and development. The following section highlights the main challenges encountered in the development of coronavirus vaccines.

### 8.1. High Mutation Rates and Viral RNA Quasispecies

Animal coronaviruses have been implicated in several outbreaks, notably SARS-CoV, MERS-CoV, and SARS-CoV-2. The formidable hurdle in vaccine development arises from the heightened mutation rates observed in these viruses, rendering the efficacy of vaccines susceptible to the presence of viral genome mutations [236]. With each mutation, different strains may exhibit varying antigenic profiles, thereby complicating the identification of specific antigens for targeted vaccine development [236]. This challenge is due to the high genetic diversity of coronaviruses, resulting from the high mutation rate and recombination events during viral replication, which generates related but distinct viral RNA sequences, known as quasispecies [237]. RNA quasispecies can cause antigenic drift, altering the effectiveness of vaccines that target specific epitopes of the virus, making it challenging to develop a vaccine that can provide long-term protection [238]. Additionally, RNA quasispecies can lead to the emergence of novel virus strains, necessitating the development of new vaccines [237,239]. Therefore, successful animal coronavirus vaccine development requires a comprehensive understanding of the viral quasispecies present in the target population, as well as the ability to predict and monitor the evolution of the virus in response to vaccine-induced immune pressure.

### 8.2. Lack of Suitable Cell Lines Capable of High Yield Production

The lack of suitable cell lines capable of producing high viral yields presents a significant challenge in the development of vaccines for animal coronaviruses. The isolation and cultivation of several animal coronaviruses in vitro have not yet been successful due to the inadequacy of suitable cell lines, which makes it arduous to isolate and study the viruses, thereby impeding vaccine development. Even in cases where cell lines, such as those utilized for the IBV, are deemed suitable, the viral yield often remains suboptimal, necessitating the addition of exogenous trypsin to the culture medium to support viral growth and propagation [240]. These circumstances underscore the considerable distance these cell lines must traverse before they can be employed for large-scale vaccine production in manufacturing facilities. Moreover, the use of serum-free culture medium for mass vaccine production is a crucial consideration from a production cost perspective. Therefore, researchers must cultivate these cell lines that are not inherently capable of virus growth and increase the speed of animal coronavirus vaccine development.

### 8.3. “Off-Target” Antibody Responses

Antibodies are an essential component of the immune response and play a vital role in neutralizing viral infections. However, antibodies can also target unintended antigens, leading to “off-target” antibody responses [241]. One challenge related to “off-target” antibody responses in animal coronavirus vaccine development is that the antibodies produced in response to a vaccine may not be specific to the target virus. Instead, the antibodies may recognize and bind to other viruses or even the body’s own cells, leading to unintended effects such as autoimmune reactions. This can be a particular concern with coronavirus vaccines, as the viruses are highly variable and can mutate rapidly, potentially leading to the production of non-specific antibodies [236,237]. Thus, developing vaccines that elicit a highly specific and effective immune response, while minimizing the risk of off-target effects, is an important challenge in animal coronavirus vaccine development. Recently, we have suggested two potential solutions to address this issue: (1) One approach is to focus on the development of vaccines that stimulate neutralizing antibody responses while minimizing the production of non-neutralizing antibodies. This approach can be achieved through the use of adjuvants that stimulate the production of neutralizing antibodies, or through the use of protein subunits that are less likely to elicit non-neutralizing antibodies; (2) Careful selection of vaccine candidates and monitoring of immune responses during clinical trials is essential to reducing the risk of “off-target” antibody responses. Vaccine candidates should be chosen based on their ability to stimulate neutralizing antibody responses while minimizing the production of non-neutralizing antibodies. Monitoring immune responses during clinical trials can provide insight into the production of non-neutralizing antibodies and guide modifications to vaccine design to reduce their production.

### 8.4. Antibody-Dependent Enhancement (ADE)

Antibody-dependent enhancement (ADE) is a phenomenon where specific virus-related antibodies, via the Fc-receptor pathway, aid the entry of viruses into host cells, causing an increase in virus infection [242,243]. While ADE may positively impact viral entry under certain conditions, it may also exacerbate clinical diseases. In the realm of vaccine development, ADE can occur when vaccine-generated antibodies are insufficient in quantity or lack neutralizing properties, resulting in heightened binding efficacy of virus-antibody complexes to cells bearing Fc receptors. Instances of ADE have been documented in vaccines targeting dengue, HIV, and coronaviruses [203,244,245]. While direct evidence of ADE in SARS-CoV-2 remains elusive, clinical investigations of SARS-CoV vaccine candidates have suggested potential disease exacerbation attributable to ADE [246,247]. For animal coronaviruses, ADE has already been observed in cats vaccinated against the FIPV, where the vaccine generated non-neutralizing antibodies that led to more severe symptoms upon FIPV exposure [248].

### 8.5. Vaccine-Associated Enhanced Diseases (VAED)

Vaccine-associated enhanced disease (VAED) is a phenomenon characterized by the exacerbation of disease following subsequent infection with the associated pathogen due to prior vaccination [249]. VAED has been documented in several viral infections, including those caused by animal coronaviruses. The underlying mechanisms driving VAED remain incompletely understood, but two potential mechanisms have been proposed. The first mechanism is antibody-dependent enhancement (ADE), where non-neutralizing antibodies elicited by vaccination can facilitate viral entry into cells, resulting in increased viral replication and disease severity. The other likely mechanism is cell-mediated immunity, where the vaccine-induced immune response may result in immunopathology upon exposure to the pathogen. In some cases, the development of animal coronavirus vaccines is hindered by VAED. However, there are strategies available to minimize these risks, such as optimizing vaccine formulations to produce neutralizing antibodies, using adjuvants to boost the immune response, and conducting thorough clinical assessments of vaccine safety and effectiveness. Through precise development of vaccines, the use of adjuvants, and comprehensive assessment of safety and efficacy, the negative effects of VAED can be successfully reduced in the creation of animal coronavirus vaccines.

### 8.6. Recombination Events between Human and Animal CoV Strains

CoVs possess a large RNA genome that is prone to frequent mutations and recombination, leading to the emergence of strains with distinct antigenic properties. Recombination events occur when different strains infect the same host cell, resulting in the creation of novel genotypes/serotypes and variants, and the exchange of genetic material [250,251]. These new strains may evade the protection offered by existing vaccines as they possess different antigenic profiles. The limited understanding of virus evolution and recombination hinders progress in coronavirus vaccine research [252]. Moreover, the increasing incidence of spillback to other animal species, as observed with SARS-CoV-2, emphasizes the need for the development of effective vaccines against animal coronaviruses [253]. The challenge of recombination events extends to animal coronavirus vaccine development, underscoring the necessity of creating vaccines that offer broad protection against multiple CoV strains. The rapid evolution and emergence of new variants in certain CoVs, such as PEDV, can compromise the effectiveness of existing vaccines by evading the immune response. To address these obstacles, one approach involves developing recombination-resistant coronaviruses as vaccines for animal coronaviruses [254]. Another strategy entails the development of broadly protective vaccines capable of conferring immunity against multiple strains, including those resulting from recombination events.

In general, creating vaccines for animal coronaviruses is a complex process that demands a deep understanding of how the virus spreads, evolves, and causes disease. It also requires substantial resources and the ability to overcome obstacles in order to move the project forward. Despite these challenges, the veterinary community has achieved major advancements in developing animal coronavirus vaccines for pigs, dogs, and cats, demonstrating that it is feasible to create effective vaccines for animal coronaviruses.

## 9. Urgent Need to Develop a “Dual-Effect” Vaccine Capable of Inducing Both Cellular and Humoral Immune Responses

In the field of immunology, cellular immunity and humoral immunity are essential components of the immune response. Cellular immunity, or cell-mediated immunity, is carried out by T cells and natural killer cells. In contrast, humoral immunity, or antibody-mediated immunity, involves the production and function of antibodies to fight off pathogens. It is important to recognize that these two types of immunity are interconnected and collaborate to defend the body against harmful invaders [255,256]. Consequently, it can be suggested that a “dual-effect” vaccine capable of stimulating both cellular and humoral immune responses holds great potential for clinical applications. A “dual-effect” animal coronavirus vaccine would be beneficial for several reasons: (1) It would provide broader protection against different viral strains. A humoral response alone is not sufficient to combat highly variable viruses such as coronaviruses because they frequently mutate, leading to antigenic variation. A cellular response is required to target conserved regions of the virus that are less likely to change during evolution. Therefore, a “dual-effect” vaccine could provide more effective protection against new and emerging strains of coronaviruses; (2) A “dual-effect” vaccine could be more cost-effective than separate vaccines. Currently, there are no licensed vaccines for many animal coronaviruses, and developing individual vaccines for each strain would be time-consuming and expensive. A single “dual-effect” vaccine could provide broad protection against multiple coronaviruses, streamlining the vaccination process; (3) It could also help reduce the risk of zoonotic transmission. Many coronaviruses infect animals, and some, including SARS-CoV-2, can jump to humans, causing severe disease. A vaccine that induces both cellular and humoral responses in animals would not only protect them but also reduce the likelihood of zoonotic transfer [256]; (4) It could lead to better herd immunity. The combination of cellular and humoral responses would create a stronger defense against viral infections, reducing the chance of an outbreak occurring and limiting its spread if it does. This would be especially important in farm settings, where animals are often housed in close quarters and can spread infections rapidly.

Hence, the ideal vaccine against coronaviruses should elicit the generation of neutralizing antibodies that impede viral attachment and entry into host cells, along with provoking cellular immune responses capable of eliminating infected cells. In light of the limited extent of research in this field, the imperative for the development of a “dual-effect” animal coronavirus vaccine that can effectively stimulate both cellular and humoral immune responses has emerged within the realm of vaccine advancement [82]. Presently, most vaccines developed for animal coronaviruses mainly stimulate the production of virus-neutralizing antibodies, resulting in humoral immune responses [257]. However, there is an increasing awareness of the significance of inducing cellular immunity to achieve optimal protection against viral infections [258]. Consequently, numerous avenues are under exploration to foster the creation of “dual-effect” vaccines, encompassing live-attenuated vaccines, inactivated vaccines, subunit vaccines, and DNA/RNA-based vaccines [259]. These approaches have exhibited promising outcomes in preclinical investigations, demonstrating their capacity to elicit robust cellular and humoral immune responses against animal coronaviruses.

## 10. Concluding Remarks and Future Perspectives

It is acknowledged that developing effective vaccines necessitates a comprehensive understanding of viral protein biology [69], and the development of animal coronavirus vaccines is critical in preventing future outbreaks and mitigating their impact on public health. Although understanding the coronavirus’s biology is fundamental to designing animal coronavirus vaccines, it is only the beginning of a long process that leads to an effective vaccine. Our understanding of pathogenic mechanisms, genetic evolution patterns, and vaccine development for different coronaviruses still has gaps, despite significant progress. To overcome these challenges, future research should focus on several areas: (1) There is a need for interdisciplinary collaboration between virologists, immunologists, and cell biologists to investigate the pathogenic mechanisms of different coronaviruses and their interactions with host cells; (2) Novel vaccine strategies that provide “one shot to prevent multiple diseases” vaccine must be developed. This means that innovative approaches such as viral vector-based vaccines, mRNA vaccines, nanotechnology vaccines, and multi-epitope vaccines must be explored; (3) Collaboration and communication among researchers and industry stakeholders must be prioritized to ensure that research findings are translated into effective interventions that can benefit both animal and human health; (4) It should be noted that the majority of CoV vaccine candidates target the spike protein, which exhibits high variability, posing a challenge in providing long-term protection against newly emerging CoV strains; (5) In order to achieve the optimal immune effect of vaccines, new vaccine delivery routes, adjuvants, and novel approaches for the design, delivery, and administration of vaccine technologies should be continuously explored and applied [260,261].

In summary, the development of animal coronavirus vaccines is crucial for controlling the spread of the virus and minimizing the threat it poses to human health. Although there have been notable advancements in vaccine research and development, there is still much to be done. Future research should focus on identifying more animal coronaviruses, developing more effective vaccine delivery methods, and improving vaccine efficacy and safety. Through continued research and collaboration, it is possible to develop vaccines that effectively control the spread of animal coronaviruses and reduce their impact on human health.

## Figures and Tables

**Table 1:** Coronavirus taxonomy, hosts, tissue tropism and clinical illness in animals and humans.

Genus	Virus Name	Host	Tissue Tropism	Cellular Receptor	Clinical Illness	Reference
Alpha	Canine coronavirus (CCoV)	Dog, Human	Intestines, respiratory tract, lungs	APN, Sialic Acid	Diarrhea, vomiting, drowsiness, mild fever, pneumonia	[1,28,29,30,31,32]
	Feline coronavirus (FCoV)	Cat	Intestines, monocytes	APN, Sialic Acid	Peritonitis, enteritis	[33]
	Transmissible gastroenteritis virus (TGEV)	Pig	Intestines	APN, Sialic Acid	Gastroenteritis	[34,35]
	Porcine respiratory coronavirus (PRCV)	Pig	Respiratory tract, lungs, tonsils	APN	Fever, dyspnea, coughing	[36,37,38]
	Porcine epidemic diarrhea virus (PEDV)	Pig	Intestines	ND	Gastroenteritis	[39,40]
	Human coronavirus 229E (HCoV-229E)	Human	Intestines, respiratory tract	APN	Colds, pneumonia	[41,42]
	Human coronavirus NL63 (HCoV-NL63)	Human	Intestines, respiratory tract, duodenum, heart, kidney	ACE2	Colds, pneumonia	[42,43,44,45]
	Swine acute diarrhea syndrome-coronavirus (SADS-CoV)	Pig	Intestines	ND	Diarrhea, vomiting	[36,37,46,47]
	Bat coronaviruses (Bat CoV)	Bat	Intestines	ACE2	Diarrhea	[48]
Beta	Bovine coronavirus (BCoV)	Cattle	Respiratory tract, intestines, trachea, lungs	Sialic Acid	Gastroenteritis, pneumonia	[29]
	Mouse hepatitis virus (MHV)	Murine	Respiratory tract, intestines, CNS	CEACAM	Hepatitis, encephalitis	[49,50]
	Porcine hemagglutinating encephalomyelitis (PHEV)	Pig	CNS	ND	Neurological and/or enteric disease	[51]
	Human coronavirus OC43 (HCoV-OC43)	Human	Intestines, respiratory tract	Sialic Acid	Colds, pneumonia	[29,33]
	Middle east respiratory syndrome coronavirus (MERS-CoV)	Camel, Human	Intestines, kidney, placenta	DPP4 (CD26)	Respiratory disease	[50,52]
	Severe acute respiratory syndrome coronavirus (SARS-CoV)	Human	Intestines, duodenum, heart, kidney	ACE2	Pneumonia, gastroenteritis	[43,53]
	Severe acute respiratory syndrome coronavirus 2 (SARS-CoV-2)	Human	Intestines, duodenum, heart, kidney	ACE2, KREMEN1, ASGR1	Pneumonia, gastroenteritis	[43,54,55,56,57]
	Equine coronavirus (ECoV)	Horse	Respiratory tract, CNS	ND	Diarrhea, fever, lethargy, and anorexia	[36,58]
Gamma	Infectious bronchitis virus (IBV)	Avian	Respiratory tract, intestines, kidney	Sialic Acid	Respiratory, kidney, oviduct, and intestinal tract disease	[36,59,60]
	Turkey coronavirus (TCoV)	Turkey	Intestines	Sialic Acid	Enteric disease	[37]
Delta	Porcine deltacoronavirus (PDCoV)	Pig, Human	Intestines	APN	Enteric disease, fever, cough, abdominal pain	[2,37,61]

Note: APN, aminopeptidase N; CEACAM, carcinoembryonic antigen-related cell adhesion molecules; ACE2, angiotensin-converting enzyme 2; DPP4, dipeptidyl peptidase 4; ASGR1, asialoglycoprotein receptor-1; KREMEN1, kringle containing transmembrane protein 1; CNS, central nervous system; ND, not determined.

**Table 2:** The types, platforms, and immunization routes of various trialed or generated animal coronavirus vaccines.

Genus	Targeted Viruses	Vaccine Name/ Candidate Strains	Vaccine Platform	Immunization Route	Reference
Alpha	CCoV	Strain 257/98-3c	Whole virus	Intramuscular (IM)	[142]
	FCoV	FIPV-DF2	Live-attenuated virus	Feline kidney (NLFK) cell/ intranasal	[143]
		FIPV 79-1146	Live-attenuated virus	FCWF/inoculated oronasally	[144]
		FIPV-M, FIPV-M (VR1012-M, VR1012-N)	DNA vaccine	Plasmid injection	[145]
	PEDV	CV777, 83P-5	Live-attenuated virus	Intramuscular (IM)	[146,147]
		SM98-1	Live-attenuated virus	Intramuscular (IM)	[148]
		DR13	Live-attenuated virus	Oral	[149]
		KNU-141112,	Inactivated virus	Intramuscular (IM)	[150]
		PEDV CO2013	Live-attenuated virus	Intramuscular (IM) and oral	[151]
		USA/Colorado/2013	Live-attenuated virus	Intragastric route	[152]
		AH2012/12	Inactivated virus	Intranasal	[153]
		MZ0116-2/2013	Live-attenuated virus	Intramuscular (IM)	[154]
		3B3scFv-pFc-PEDVsAg	DNA vaccine	Intramuscular (IM)	[155]
		ORFV-PEDV-S	Recombinant virus	Intramuscular (IM)	[156]
		S mRNA-LNP vaccine/ Sm mRNA-LNP	mRNA vaccine	Intramuscular (IM)	[157]
		pTriEx-S (S1)	DNA vaccine	Intramuscular (IM)	[158]
		rSF-COE-3D	Recombinant virus	Intramuscular (IM)	[159]
		rTGEV-RS-SPEDV	Recombinant virus	Oral	[160]
	TGEV	TGE/Rota	Recombinant virus	Intramuscular (IM), oral	[38]
Beta	BCoV	438/06-TN	Live-attenuated virus	Intramuscular (IM)	[161]
	MHV	TMV-5B19	DNA vaccine	Intranasal or subcutaneous	[162]
Gamma	IBV	K2/01	Live-attenuated virus	Eye drop	[163]
		pCAG-N	DNA vaccine	Intranasal (IN)	[164]
Delta	PDCoV	L. lactis NZ9000-S1	DNA vaccine	Oral	[165]
		CZ2020	Live-attenuated virus	Oral	[166]
		PDCoV-NH	Inactivated virus	Oral	[61]

## Data Availability

Data sharing is not applicable to this article.

## References

[1] Vlasova A.N., Diaz A., Damtie D., Xiu L., Toh T.-H., Lee J.S.-Y., Saif L.J., Gray G.C. (2021). Novel Canine Coronavirus Isolated from a Hospitalized Patient With Pneumonia in East Malaysia. Clin. Infect. Dis..

[2] Lednicky J.A., Tagliamonte M.S., White S.K., Elbadry M.A., Alam M.M., Stephenson C.J., Bonny T.S., Loeb J.C., Telisma T., Chavannes S. (2021). Independent infections of porcine deltacoronavirus among Haitian children. Nature.

[3] Cui J., Li F., Shi Z.-L. (2019). Origin and evolution of pathogenic coronaviruses. Nat. Rev. Microbiol..

[4] Chen B., Tian E.-K., He B., Tian L., Han R., Wang S., Xiang Q., Zhang S., El Arnaout T., Cheng W. (2020). Overview of lethal human coronaviruses. Signal Transduct. Target. Ther..

[5] Woo P.C., Lau S.K., Lam C.S., Lau C.C., Tsang A.K., Lau J.H., Bai R., Teng J.L., Tsang C.C., Wang M. (2012). Discovery of seven novel Mammalian and avian coronaviruses in the genus deltacoronavirus supports bat coronaviruses as the gene source of alphacoronavirus and betacoronavirus and avian coronaviruses as the gene source of gammacoronavirus and deltacoronavirus. J. Virol..

[6] Ge X.-Y., Li J.-L., Yang X.-L., Chmura A.A., Zhu G., Epstein J.H., Mazet J.K., Hu B., Zhang W., Peng C. (2013). Isolation and characterization of a bat SARS-like coronavirus that uses the ACE2 receptor. Nature.

[7] Ge X.-Y., Yang W.-H., Zhou J.-H., Li B., Zhang W., Shi Z.-L., Zhang Y.-Z. (2017). Detection of alpha-and betacoronaviruses in rodents from Yunnan, China. Virol. J..

[8] Hu B., Zeng L.-P., Yang X.-L., Ge X.-Y., Zhang W., Li B., Xie J.-Z., Shen X.-R., Zhang Y.-Z., Wang N. (2017). Discovery of a rich gene pool of bat SARS-related coronaviruses provides new insights into the origin of SARS coronavirus. PLoS Pathog..

[9] Li W., Shi Z., Yu M., Ren W., Smith C., Epstein J.H., Wang H., Crameri G., Hu Z., Zhang H. (2005). Bats are natural reservoirs of SARS-like coronaviruses. Science.

[10] Hu B., Ge X., Wang L.-F., Shi Z. (2015). Bat origin of human coronaviruses. Virol. J..

[11] Cherry J.D., Krogstad P. (2004). SARS: The first pandemic of the 21st century. Pediatr. Res..

[12] Corman V.M., Ithete N.L., Richards L.R., Schoeman M.C., Preiser W., Drosten C., Drexler J.F. (2014). Rooting the phylogenetic tree of middle East respiratory syndrome coronavirus by characterization of a conspecific virus from an African bat. J. Virol..

[13] V’kovski P., Kratzel A., Steiner S., Stalder H., Thiel V. (2021). Coronavirus biology and replication: Implications for SARS-CoV-2. Nat. Rev. Microbiol..

[14] Wacharapluesadee S., Tan C.W., Maneeorn P., Duengkae P., Zhu F., Joyjinda Y., Kaewpom T., Chia W.N., Ampoot W., Lim B.L. (2021). Evidence for SARS-CoV-2 related coronaviruses circulating in bats and pangolins in Southeast Asia. Nat. Commun..

[15] Worobey M., Levy J.I., Malpica Serrano L., Crits-Christoph A., Pekar J.E., Goldstein S.A., Rasmussen A.L., Kraemer M.U.G., Newman C., Koopmans M.P.G. (2022). The Huanan Seafood Wholesale Market in Wuhan was the early epicenter of the COVID-19 pandemic. Science.

[16] Vijgen L., Keyaerts E., Moës E., Thoelen I., Wollants E., Lemey P., Vandamme A.-M., Van Ranst M. (2005). Complete genomic sequence of human coronavirus OC43: Molecular clock analysis suggests a relatively recent zoonotic coronavirus transmission event. J. Virol..

[17] Corman V.M., Baldwin H.J., Tateno A.F., Zerbinati R.M., Annan A., Owusu M., Nkrumah E.E., Maganga G.D., Oppong S., Adu-Sarkodie Y. (2015). Evidence for an Ancestral Association of Human Coronavirus 229E with Bats. J. Virol..

[18] Tao Y., Shi M., Chommanard C., Queen K., Zhang J., Markotter W., Kuzmin I.V., Holmes E.C., Tong S. (2017). Surveillance of Bat Coronaviruses in Kenya Identifies Relatives of Human Coronaviruses NL63 and 229E and Their Recombination History. J. Virol..

[19] Graham R.L., Donaldson E.F., Baric R.S. (2013). A decade after SARS: Strategies for controlling emerging coronaviruses. Nat. Rev. Microbiol..

[20] Cankat S., Demael M.U., Swadling L. (2024). In search of a pan-coronavirus vaccine: Next-generation vaccine design and immune mechanisms. Cell. Mol. Immunol..

[21] Tan C.W., Valkenburg S.A., Poon L.L., Wang L.-F. (2023). Broad-spectrum pan-genus and pan-family virus vaccines. Cell Host Microbe.

[22] Woo P.C.Y., de Groot R.J., Haagmans B., Lau S.K.P., Neuman B.W., Perlman S., Sola I., van der Hoek L., Wong A.C.P., Yeh S.-H. (2023). ICTV Virus Taxonomy Profile: Coronaviridae 2023. J. Gen. Virol..

[23] Zhou Z., Qiu Y., Ge X. (2021). The taxonomy, host range and pathogenicity of coronaviruses and other viruses in the Nidovirales order. Anim. Dis..

[24] Hussein H.A., Hassan R.Y., Chino M., Febbraio F. (2020). Point-of-care diagnostics of COVID-19: From current work to future perspectives. Sensors.

[25] Bukhari K., Mulley G., Gulyaeva A.A., Zhao L., Shu G., Jiang J., Neuman B.W. (2018). Description and initial characterization of metatranscriptomic nidovirus-like genomes from the proposed new family Abyssoviridae, and from a sister group to the Coronavirinae, the proposed genus Alphaletovirus. Virology.

[26] MacLachlan N.J., Dubovi E.J. (2017). Chapter 24—Coronaviridae. Fenner’s Veterinary Virology.

[27] Decaro N., Lorusso A. (2020). Novel human coronavirus (SARS-CoV-2): A lesson from animal coronaviruses. Vet. Microbiol..

[28] Regan A.D., Millet J.K., Tse L.P.V., Chillag Z., Rinaldi V.D., Licitra B.N., Dubovi E.J., Town C.D., Whittaker G.R. (2012). Characterization of a recombinant canine coronavirus with a distinct receptor-binding (S1) domain. Virology.

[29] Szczepanski A., Owczarek K., Bzowska M., Gula K., Drebot I., Ochman M., Maksym B., Rajfur Z., Mitchell J.A., Pyrc K. (2019). Canine respiratory coronavirus, bovine coronavirus, and human coronavirus OC43: Receptors and attachment factors. Viruses.

[30] Lednicky J.A., Tagliamonte M.S., White S.K., Blohm G.M., Alam M.M., Iovine N.M., Salemi M., Mavian C., Morris J.G. (2022). Isolation of a novel recombinant canine coronavirus from a visitor to Haiti: Further evidence of transmission of coronaviruses of zoonotic origin to humans. Clin. Infect. Dis..

[31] Tortorici M.A., Walls A.C., Joshi A., Park Y.-J., Eguia R.T., Miranda M.C., Kepl E., Dosey A., Stevens-Ayers T., Boeckh M.J. (2022). Structure, receptor recognition, and antigenicity of the human coronavirus CCoV-HuPn-2018 spike glycoprotein. Cell.

[32] Liu Y., Chen D., Wang Y., Li X., Qiu Y., Zheng M., Song Y., Li G., Song C., Liu T. (2023). Characterization of CCoV-HuPn-2018 spike protein-mediated viral entry. J. Virol..

[33] Hulswit R.J., Lang Y., Bakkers M.J., Li W., Li Z., Schouten A., Ophorst B., Van Kuppeveld F.J., Boons G.-J., Bosch B.-J. (2019). Human coronaviruses OC43 and HKU1 bind to 9-O-acetylated sialic acids via a conserved receptor-binding site in spike protein domain A. Proc. Natl. Acad. Sci. USA.

[34] Delmas B., Gelfi J., L’Haridon R., Sjöström H., Laude H. (1992). Aminopeptidase N is a major receptor for the enteropathogenic coronavirus TGEV. Nature.

[35] Chen Y., Zhang Y., Wang X., Zhou J., Ma L., Li J., Yang L., Ouyang H., Yuan H., Pang D. (2023). Transmissible Gastroenteritis Virus: An Update Review and Perspective. Viruses.

[36] Liu Q., Wang H.-Y. (2021). Porcine enteric coronaviruses: An updated overview of the pathogenesis, prevalence, and diagnosis. Vet. Res. Commun..

[37] Islam A., Ferdous J., Islam S., Sayeed M., Dutta Choudhury S., Saha O., Hassan M.M., Shirin T. (2021). Evolutionary dynamics and epidemiology of endemic and emerging coronaviruses in humans, domestic animals, and wildlife. Viruses.

[38] Turlewicz-Podbielska H., Pomorska-Mól M. (2021). Porcine coronaviruses: Overview of the state of the art. Virol. Sin..

[39] Lin F., Zhang H., Li L., Yang Y., Zou X., Chen J., Tang X. (2022). PEDV: Insights and Advances into Types, Function, Structure, and Receptor Recognition. Viruses.

[40] Liu C., Tang J., Ma Y., Liang X., Yang Y., Peng G., Qi Q., Jiang S., Li J., Du L. (2015). Receptor usage and cell entry of porcine epidemic diarrhea coronavirus. J. Virol..

[41] Yeager C.L., Ashmun R.A., Williams R.K., Cardellichio C.B., Shapiro L.H., Look A.T., Holmes K.V. (1992). Human aminopeptidase N is a receptor for human coronavirus 229E. Nature.

[42] Liu D., Chen C., Chen D., Zhu A., Li F., Zhuang Z., Mok C.K.P., Dai J., Li X., Jin Y. (2023). Mouse models susceptible to HCoV-229E and HCoV-NL63 and cross protection from challenge with SARS-CoV-2. Proc. Natl. Acad. Sci. USA.

[43] Wan Y., Shang J., Graham R., Baric R.S., Li F. (2020). Receptor recognition by the novel coronavirus from Wuhan: An analysis based on decade-long structural studies of SARS coronavirus. J. Virol..

[44] Wang Y., Li X., Liu W., Gan M., Zhang L., Wang J., Zhang Z., Zhu A., Li F., Sun J. (2020). Discovery of a subgenotype of human coronavirus NL63 associated with severe lower respiratory tract infection in China, 2018. Emerg. Microbes Infect..

[45] Hofmann H., Pyrc K., Van Der Hoek L., Geier M., Berkhout B., Pöhlmann S. (2005). Human coronavirus NL63 employs the severe acute respiratory syndrome coronavirus receptor for cellular entry. Proc. Natl. Acad. Sci. USA.

[46] Yang Y.-L., Yu J.-Q., Huang Y.-W. (2020). Swine enteric alphacoronavirus (swine acute diarrhea syndrome coronavirus): An update three years after its discovery. Virus Res..

[47] Yu D., Zhao Z.-Y., Yang Y.-L., Qin Y., Pan D., Yuan L.-X., Huang Y.-W., Wang B. (2023). The origin and evolution of emerged swine acute diarrhea syndrome coronavirus with zoonotic potential. J. Med. Virol..

[48] Guo H., Hu B., Si H.-R., Zhu Y., Zhang W., Li B., Li A., Geng R., Lin H.-F., Yang X.-L. (2021). Identification of a novel lineage bat SARS-related coronaviruses that use bat ACE2 receptor. Emerg. Microbes Infect..

[49] Hemmila E., Turbide C., Olson M., Jothy S., Holmes K.V., Beauchemin N. (2004). Ceacam1a^−/−^ Mice Are Completely Resistant to Infection by Murine Coronavirus Mouse Hepatitis Virus A59. J. Virol..

[50] Li Q., Shah T., Wang B., Qu L., Wang R., Hou Y., Baloch Z., Xia X. (2023). Cross-species transmission, evolution and zoonotic potential of coronaviruses. Front. Cell. Infect. Microbiol..

[51] Saif L.J., Jung K. (2020). Comparative pathogenesis of bovine and porcine respiratory coronaviruses in the animal host species and SARS-CoV-2 in humans. J. Clin. Microbiol..

[52] Ng L., Wong S.K.-M., Huang Z., Lam C.S.-C., Chow A.K.-M., Foo D.C.-C., Lo O.S.-H., Pang R.W.-C., Law W.-L. (2022). CD26 induces colorectal cancer angiogenesis and metastasis through CAV1/MMP1 signaling. Int. J. Mol. Sci..

[53] Li W., Moore M.J., Vasilieva N., Sui J., Wong S.K., Berne M.A., Somasundaran M., Sullivan J.L., Luzuriaga K., Greenough T.C. (2003). Angiotensin-converting enzyme 2 is a functional receptor for the SARS coronavirus. Nature.

[54] Hoffmann M., Pöhlmann S. (2022). Novel SARS-CoV-2 receptors: Asgr1 and Kremen1. Cell Res..

[55] Zamorano Cuervo N., Grandvaux N. (2020). ACE2: Evidence of role as entry receptor for SARS-CoV-2 and implications in comorbidities. eLife.

[56] Hoffmann M., Kleine-Weber H., Schroeder S., Krüger N., Herrler T., Erichsen S., Schiergens T.S., Herrler G., Wu N.-H., Nitsche A. (2020). SARS-CoV-2 cell entry depends on ACE2 and TMPRSS2 and is blocked by a clinically proven protease inhibitor. Cell.

[57] Gu Y., Cao J., Zhang X., Gao H., Wang Y., Wang J., He J., Jiang X., Zhang J., Shen G. (2022). Receptome profiling identifies KREMEN1 and ASGR1 as alternative functional receptors of SARS-CoV-2. Cell Res..

[58] Pusterla N., Vin R., Leutenegger C.M., Mittel L.D., Divers T.J. (2018). Enteric coronavirus infection in adult horses. Vet. J..

[59] Winter C., Schwegmann-Weßels C., Cavanagh D., Neumann U., Herrler G. (2006). Sialic acid is a receptor determinant for infection of cells by avian Infectious bronchitis virus. J. Gen. Virol..

[60] You R., Liu K., Huang M., Tang L., Zhang X., Huang Y., Zhao J., Zhao Y., Ye L., Zhang G. (2023). Identification and Comparison of the Sialic Acid-Binding Domain Characteristics of Avian Coronavirus Infectious Bronchitis Virus Spike Protein. J. Virol..

[61] Zhang J., Chen J., Liu Y., Da S., Shi H., Zhang X., Liu J., Cao L., Zhu X., Wang X. (2020). Pathogenicity of porcine deltacoronavirus (PDCoV) strain NH and immunization of pregnant sows with an inactivated PDCoV vaccine protects 5-day-old neonatal piglets from virulent challenge. Transbound. Emerg. Dis..

[62] Wang Q., Vlasova A.N., Kenney S.P., Saif L.J. (2019). Emerging and re-emerging coronaviruses in pigs. Curr. Opin. Virol..

[63] Kenney S.P., Wang Q., Vlasova A., Jung K., Saif L. (2021). Naturally occurring animal coronaviruses as models for studying highly pathogenic human coronaviral disease. Vet. Pathol..

[64] Khamassi Khbou M., Daaloul Jedidi M., Bouaicha Zaafouri F., Benzarti M.h. (2021). Coronaviruses in farm animals: Epidemiology and public health implications. Vet. Med. Sci..

[65] Gerdts V., Zakhartchouk A. (2017). Vaccines for porcine epidemic diarrhea virus and other swine coronaviruses. Vet. Microbiol..

[66] Stevenson G.W., Hoang H., Schwartz K.J., Burrough E.R., Sun D., Madson D., Cooper V.L., Pillatzki A., Gauger P., Schmitt B.J. (2013). Emergence of Porcine epidemic diarrhea virus in the United States: Clinical signs, lesions, and viral genomic sequences. J. Vet. Diagn. Investig..

[67] Li W., van Kuppeveld F.J., He Q., Rottier P.J., Bosch B.-J. (2016). Cellular entry of the porcine epidemic diarrhea virus. Virus Res..

[68] Si F., Chen B., Hu X., Yu R., Dong S., Wang R., Li Z. (2020). Porcine Epidemic Diarrhea Virus ORF3 Protein Is Transported through the Exocytic Pathway. J. Virol..

[69] Si F., Song S., Yu R., Li Z., Wei W., Wu C. (2023). Coronavirus accessory protein ORF3 biology and its contribution to viral behavior and pathogenesis. iScience.

[70] Zhou Y., Zhang Y., Dong W., Gan S., Du J., Zhou X., Fang W., Wang X., Song H. (2023). Porcine epidemic diarrhea virus activates PERK-ROS axis to benefit its replication in Vero E6 cells. Vet. Res..

[71] Wang H., Kong N., Jiao Y., Dong S., Sun D., Chen X., Zheng H., Tong W., Yu H., Yu L. (2021). EGR1 Suppresses Porcine Epidemic Diarrhea Virus Replication by Regulating IRAV To Degrade Viral Nucleocapsid Protein. J. Virol..

[72] Doyle L., Hutchings L. (1946). A transmissible gastroenteritis in pigs. J. Am. Vet. Med. Assoc..

[73] Lamphear B.J., Jilka J.M., Kesl L., Welter M., Howard J.A., Streatfield S.J. (2004). A corn-based delivery system for animal vaccines: An oral transmissible gastroenteritis virus vaccine boosts lactogenic immunity in swine. Vaccine.

[74] Chattha K.S., Roth J.A., Saif L.J. (2015). Strategies for Design and Application of Enteric Viral Vaccines. Annu. Rev. Anim. Biosci..

[75] Moxley R., Olson L. (1989). Clinical evaluation of transmissible gastroenteritis virus vaccines and vaccination procedures for inducing lactogenic immunity in sows. Am. J. Vet. Res..

[76] Bohl E.H., Gupta R.K.P., Olquin M.V.F., Saif L.J. (1972). Antibody Responses in Serum, Colostrum, and Milk of Swine After Infection or Vaccination with Transmissible Gastroenteritis Virus. Infect. Immun..

[77] Mora-Díaz J.C., Piñeyro P.E., Houston E., Zimmerman J., Giménez-Lirola L.G. (2019). Porcine hemagglutinating encephalomyelitis virus: A review. Front. Vet. Sci..

[78] Li Z., He W., Lan Y., Zhao K., Lv X., Lu H., Ding N., Zhang J., Shi J., Shan C. (2016). The evidence of porcine hemagglutinating encephalomyelitis virus induced nonsuppurative encephalitis as the cause of death in piglets. PeerJ.

[79] Li W., Hulswit R.J., Kenney S.P., Widjaja I., Jung K., Alhamo M.A., van Dieren B., van Kuppeveld F.J., Saif L.J., Bosch B.-J. (2018). Broad receptor engagement of an emerging global coronavirus may potentiate its diverse cross-species transmissibility. Proc. Natl. Acad. Sci. USA.

[80] Ji W., Peng Q., Fang X., Li Z., Li Y., Xu C., Zhao S., Li J., Chen R., Mo G. (2022). Structures of a deltacoronavirus spike protein bound to porcine and human receptors. Nat. Commun..

[81] Niu Z., Zhang S., Xu S., Wang J., Wang S., Hu X., Zhang L., Ren L., Zhang J., Liu X. (2023). Porcine Epidemic Diarrhea Virus Replication in Human Intestinal Cells Reveals Potential Susceptibility to Cross-Species Infection. Viruses.

[82] Alluwaimi A.M., Alshubaith I.H., Al-Ali A.M., Abohelaika S. (2020). The coronaviruses of animals and birds: Their zoonosis, vaccines, and models for SARS-CoV and SARS-CoV2. Front. Vet. Sci..

[83] Pratelli A., Martella V., Decaro N., Tinelli A., Camero M., Cirone F., Elia G., Cavalli A., Corrente M., Greco G. (2003). Genetic diversity of a canine coronavirus detected in pups with diarrhoea in Italy. J. Virol. Methods.

[84] Binn L., Lazar E., Keenan K., Huxsoll D., Marchwicki R., Strano A. (1974). Recovery and characterization of a coronavirus from military dogs with diarrhea. Proc. Annu. Meet. U. S. Anim. Health Assoc..

[85] Decaro N., Buonavoglia C. (2011). Canine coronavirus: Not only an enteric pathogen. Vet. Clin. Small Anim. Pract..

[86] Buonavoglia C., Decaro N., Martella V., Elia G., Campolo M., Desario C., Castagnaro M., Tempesta M. (2006). Canine coronavirus highly pathogenic for dogs. Emerg. Infect. Dis..

[87] Decaro N., Mari V., Campolo M., Lorusso A., Camero M., Elia G., Martella V., Cordioli P., Enjuanes L., Buonavoglia C. (2009). Recombinant canine coronaviruses related to transmissible gastroenteritis virus of swine are circulating in dogs. J. Virol..

[88] Lorusso A., Desario C., Mari V., Campolo M., Lorusso E., Elia G., Martella V., Buonavoglia C., Decaro N. (2009). Molecular characterization of a canine respiratory coronavirus strain detected in Italy. Virus Res..

[89] Lu S., Chen Y., Qin K., Zhou J., Lou Y., Tan W. (2016). Genetic and antigenic characterization of recombinant nucleocapsid proteins derived from canine coronavirus and canine respiratory coronavirus in China. Sci. China Life Sci..

[90] Guy J.S., Breslin J.J., Breuhaus B., Vivrette S., Smith L.G. (2000). Characterization of a coronavirus isolated from a diarrheic foal. J. Clin. Microbiol..

[91] Mattei D.N., Kopper J.J., Sanz M.G. (2020). Equine coronavirus-associated colitis in horses: A retrospective study. J. Equine Vet. Sci..

[92] Ward J.M. (1970). Morphogenesis of a virus in cats with experimental feline infectious peritonitis. Virology.

[93] Tresnan D.B., Levis R., Holmes K.V. (1996). Feline aminopeptidase N serves as a receptor for feline, canine, porcine, and human coronaviruses in serogroup I. J. Virol..

[94] Vogel L., Van der Lubben M., Te Lintelo E.G., Bekker C.P., Geerts T., Schuijff L.S., Grinwis G.C., Egberink H.F., Rottier P.J. (2010). Pathogenic characteristics of persistent feline enteric coronavirus infection in cats. Vet. Res..

[95] Wang Y.-T., Su B.-L., Hsieh L.-E., Chueh L.-L. (2013). An outbreak of feline infectious peritonitis in a Taiwanese shelter: Epidemiologic and molecular evidence for horizontal transmission of a novel type II feline coronavirus. Vet. Res..

[96] Kipar A., Meli M. (2014). Feline infectious peritonitis: Still an enigma?. Vet. Pathol..

[97] Felten S., Hartmann K. (2019). Diagnosis of feline infectious peritonitis: A review of the current literature. Viruses.

[98] Day M.J., Horzinek M., Schultz R., Squires R. (2016). WSAVA Guidelines for the vaccination of dogs and cats. J. Small Anim. Pract..

[99] Benfield D., Saif L. (1990). Cell culture propagation of a coronavirus isolated from cows with winter dysentery. J. Clin. Microbiol..

[100] Saif L.J. (2010). Bovine respiratory coronavirus. Vet. Clin. Food Anim. Pract..

[101] Hasoksuz M., Alekseev K., Vlasova A., Zhang X., Spiro D., Halpin R., Wang S., Ghedin E., Saif L.J. (2007). Biologic, antigenic, and full-length genomic characterization of a bovine-like coronavirus isolated from a giraffe. J. Virol..

[102] Ismail M., Cho K., Ward L., Saif L., Saif Y. (2001). Experimental bovine coronavirus in turkey poults and young chickens. Avian Dis..

[103] Kaneshima T., Hohdatsu T., Hagino R., Hosoya S., Nojiri Y., Murata M., Takano T., Tanabe M., Tsunemitsu H., Koyama H. (2007). The infectivity and pathogenicity of a group 2 bovine coronavirus in pups. J. Vet. Med. Sci..

[104] Nemoto M., Kanno T., Bannai H., Tsujimura K., Yamanaka T., Kokado H. (2017). Antibody response to equine coronavirus in horses inoculated with a bovine coronavirus vaccine. J. Vet. Med. Sci..

[105] Franzo G., Legnardi M., Tucciarone C.M., Drigo M., Martini M., Cecchinato M. (2019). Evolution of infectious bronchitis virus in the field after homologous vaccination introduction. Vet. Res..

[106] Zhao Y., Zhang H., Zhao J., Zhong Q., Jin J.-h., Zhang G.-z. (2016). Evolution of infectious bronchitis virus in China over the past two decades. J. Gen. Virol..

[107] Li Y.T., Chen T.C., Lin S.Y., Mase M., Murakami S., Horimoto T., Chen H.W. (2020). Emerging lethal infectious bronchitis coronavirus variants with multiorgan tropism. Transbound. Emerg. Dis..

[108] Cavanagh D. (2007). Coronavirus avian infectious bronchitis virus. Vet. Res..

[109] Shahwan K., Hesse M., Mork A.-K., Herrler G., Winter C. (2013). Sialic acid binding properties of soluble coronavirus spike (S1) proteins: Differences between infectious bronchitis virus and transmissible gastroenteritis virus. Viruses.

[110] De Wit J., Cook J.K. (2020). Spotlight on avian coronaviruses. Avian Pathol..

[111] Liais E., Croville G., Mariette J., Delverdier M., Lucas M.-N., Klopp C., Lluch J., Donnadieu C., Guy J., Corrand L. (2014). Novel Avian Coronavirus and Fulminating Disease in Guinea Fowl, France. Emerg. Infect. Dis. J..

[112] Baron M.D., Iqbal M., Nair V. (2018). Recent advances in viral vectors in veterinary vaccinology. Curr. Opin. Virol..

[113] Laconi A., Weerts E., Bloodgood J., Marrero J.D., Berends A., Cocciolo G., de Wit J., Verheije M. (2020). Attenuated live infectious bronchitis virus QX vaccine disseminates slowly to target organs distant from the site of inoculation. Vaccine.

[114] Masoudi S., Pishraft-Sabet L., Shahsavandi S. (2020). Immunogenicity and efficacy of live infectious bronchitis 793/B. 08IR vaccine in SPF chickens. Arch. Razi Inst..

[115] Fehr A.R., Perlman S. (2015). Coronaviruses: An overview of their replication and pathogenesis. Coronaviruses: Methods and Protocols.

[116] Jaimes J.A., Whittaker G.R. (2018). Feline coronavirus: Insights into viral pathogenesis based on the spike protein structure and function. Virology.

[117] Bredenbeek P.J., Pachuk C.J., Noten A.F., Charité J., Luytjes W., Weiss S.R., Spaan W.J. (1990). The primary structure and expression of the second open reading frame of the polymerase gene of the coronavirus MHV-A59; a highly conserved polymerase is expressed by an efficient ribosomal frameshifting mechanism. Nucleic Acids Res..

[118] Lang Y., Li W., Li Z., Koerhuis D., Van Den Burg A.C., Rozemuller E., Bosch B.-J., Van Kuppeveld F.J., Boons G.-J., Huizinga E.G. (2020). Coronavirus hemagglutinin-esterase and spike proteins coevolve for functional balance and optimal virion avidity. Proc. Natl. Acad. Sci. USA.

[119] Yan W., Zheng Y., Zeng X., He B., Cheng W. (2022). Structural biology of SARS-CoV-2: Open the door for novel therapies. Signal Transduct. Target. Ther..

[120] Yurkovetskiy L., Wang X., Pascal K.E., Tomkins-Tinch C., Nyalile T.P., Wang Y., Baum A., Diehl W.E., Dauphin A., Carbone C. (2020). Structural and functional analysis of the D614G SARS-CoV-2 spike protein variant. Cell.

[121] McCallum M., De Marco A., Lempp F.A., Tortorici M.A., Pinto D., Walls A.C., Beltramello M., Chen A., Liu Z., Zatta F. (2021). N-terminal domain antigenic mapping reveals a site of vulnerability for SARS-CoV-2. Cell.

[122] Jackson C.B., Farzan M., Chen B., Choe H. (2022). Mechanisms of SARS-CoV-2 entry into cells. Nat. Rev. Mol. Cell Biol..

[123] Markov P.V., Ghafari M., Beer M., Lythgoe K., Simmonds P., Stilianakis N.I., Katzourakis A. (2023). The evolution of SARS-CoV-2. Nat. Rev. Microbiol..

[124] Gorkhali R., Koirala P., Rijal S., Mainali A., Baral A., Bhattarai H.K. (2021). Structure and function of major SARS-CoV-2 and SARS-CoV proteins. Bioinform. Biol. Insights.

[125] Wu W., Cheng Y., Zhou H., Sun C., Zhang S. (2023). The SARS-CoV-2 nucleocapsid protein: Its role in the viral life cycle, structure and functions, and use as a potential target in the development of vaccines and diagnostics. Virol. J..

[126] Pan P., Ge W., Lei Z., Luo W., Liu Y., Guan Z., Chen L., Yu Z., Shen M., Hu D. (2023). SARS-CoV-2 N protein enhances the anti-apoptotic activity of MCL-1 to promote viral replication. Signal Transduct. Target. Ther..

[127] Zhang Z., Nomura N., Muramoto Y., Ekimoto T., Uemura T., Liu K., Yui M., Kono N., Aoki J., Ikeguchi M. (2022). Structure of SARS-CoV-2 membrane protein essential for virus assembly. Nat. Commun..

[128] Mahtarin R., Islam S., Islam M.J., Ullah M.O., Ali M.A., Halim M.A. (2022). Structure and dynamics of membrane protein in SARS-CoV-2. J. Biomol. Struct. Dyn..

[129] Cao Y., Yang R., Lee I., Zhang W., Sun J., Wang W., Meng X. (2021). Characterization of the SARS-CoV-2 E protein: Sequence, structure, viroporin, and inhibitors. Protein Sci..

[130] Tomar P.P.S., Arkin I.T. (2020). SARS-CoV-2 E protein is a potential ion channel that can be inhibited by Gliclazide and Memantine. Biochem. Biophys. Res. Commun..

[131] Fang P., Fang L., Zhang H., Xia S., Xiao S. (2021). Functions of Coronavirus Accessory Proteins: Overview of the State of the Art. Viruses.

[132] Hassan S.S., Choudhury P.P., Dayhoff G.W., Aljabali A.A.A., Uhal B.D., Lundstrom K., Rezaei N., Pizzol D., Adadi P., Lal A. (2022). The importance of accessory protein variants in the pathogenicity of SARS-CoV-2. Arch. Biochem. Biophys..

[133] Redondo N., Zaldívar-López S., Garrido J.J., Montoya M. (2021). SARS-CoV-2 Accessory Proteins in Viral Pathogenesis: Knowns and Unknowns. Front. Immunol..

[134] Qu Y., Wang X., Zhu Y., Wang W., Wang Y., Hu G., Liu C., Li J., Ren S., Xiao M.Z.X. (2021). ORF3a-Mediated Incomplete Autophagy Facilitates Severe Acute Respiratory Syndrome Coronavirus-2 Replication. Front. Cell Dev. Biol..

[135] Si F., Hu X., Wang C., Chen B., Wang R., Dong S., Yu R., Li Z. (2020). Porcine Epidemic Diarrhea Virus (PEDV) ORF3 Enhances Viral Proliferation by Inhibiting Apoptosis of Infected Cells. Viruses.

[136] Piñeyro P.E., Lozada M.I., Alarcón L.V., Sanguinetti R., Cappuccio J.A., Pérez E.M., Vannucci F., Armocida A., Madson D.M., Perfumo C.J. (2018). First retrospective studies with etiological confirmation of porcine transmissible gastroenteritis virus infection in Argentina. BMC Vet. Res..

[137] Huang Y.W., Dickerman A.W., Pineyro P., Li L., Fang L., Kiehne R., Opriessnig T., Meng X.J. (2013). Origin, evolution, and genotyping of emergent porcine epidemic diarrhea virus strains in the United States. MBio.

[138] Gong L., Li J., Zhou Q., Xu Z., Chen L., Zhang Y., Xue C., Wen Z., Cao Y. (2017). A new bat-HKU2–like coronavirus in swine, China, 2017. Emerg. Infect. Dis..

[139] Pan Y., Tian X., Qin P., Wang B., Zhao P., Yang Y.-L., Wang L., Wang D., Song Y., Zhang X. (2017). Discovery of a novel swine enteric alphacoronavirus (SeACoV) in southern China. Vet. Microbiol..

[140] Zhou P., Fan H., Lan T., Yang X.-L., Shi W.-F., Zhang W., Zhu Y., Zhang Y.-W., Xie Q.-M., Mani S. (2018). Fatal swine acute diarrhoea syndrome caused by an HKU2-related coronavirus of bat origin. Nature.

[141] Sparrer McKenzie N., Hodges Natasha F., Sherman T., VandeWoude S., Bosco-Lauth Angela M., Mayo Christie E. (2023). Role of Spillover and Spillback in SARS-CoV-2 Transmission and the Importance of One Health in Understanding the Dynamics of the COVID-19 Pandemic. J. Clin. Microbiol..

[142] Pratelli A., Tinelli A., Decaro N., Martella V., Camero M., Tempesta M., Martini M., Carmichael L.E., Buonavoglia C. (2004). Safety and efficacy of a modified-live canine coronavirus vaccine in dogs. Vet. Microbiol..

[143] Fehr D., Holznagel E., Bolla S., Hauser B., Herrewegh A.A., Horzinek M.C., Lutz H. (1997). Placebo-controlled evaluation of a modified life virus vaccine against feline infectious peritonitis: Safety and efficacy under field conditions. Vaccine.

[144] Haijema B.J., Volders H., Rottier P.J. (2004). Live, attenuated coronavirus vaccines through the directed deletion of group-specific genes provide protection against feline infectious peritonitis. J. Virol..

[145] Glansbeek H.L., Haagmans B.L., te Lintelo E.G., Egberink H.F., Duquesne V., Aubert A., Horzinek M.C., Rottier P.J.M. (2002). Adverse effects of feline IL-12 during DNA vaccination against feline infectious peritonitis virus. J. Gen. Virol..

[146] De Arriba M., Carvajal A., Pozo J., Rubio P. (2002). Mucosal and systemic isotype-specific antibody responses and protection in conventional pigs exposed to virulent or attenuated porcine epidemic diarrhoea virus. Vet. Immunol. Immunopathol..

[147] Sato T., Takeyama N., Katsumata A., Tuchiya K., Kodama T., Kusanagi K.-i. (2011). Mutations in the spike gene of porcine epidemic diarrhea virus associated with growth adaptation in vitro and attenuation of virulence in vivo. Virus Genes.

[148] Kweon C.H., Kwon B.J., Lee J.G., Kwon G.O., Kang Y.B. (1999). Derivation of attenuated porcine epidemic diarrhea virus (PEDV) as vaccine candidate. Vaccine.

[149] Song D.S., Oh J.S., Kang B.K., Yang J.S., Moon H.J., Yoo H.S., Jang Y.S., Park B.K. (2007). Oral efficacy of Vero cell attenuated porcine epidemic diarrhea virus DR13 strain. Res. Vet. Sci..

[150] Baek P.-S., Choi H.-W., Lee S., Yoon I.-J., Lee Y.J., Lee D.S., Lee S., Lee C. (2016). Efficacy of an inactivated genotype 2b porcine epidemic diarrhea virus vaccine in neonatal piglets. Vet. Immunol. Immunopathol..

[151] Singh G., Singh P., Pillatzki A., Nelson E., Webb B., Dillberger-Lawson S., Ramamoorthy S. (2019). A minimally replicative vaccine protects vaccinated piglets against challenge with the porcine epidemic diarrhea virus. Front. Vet. Sci..

[152] Krishna V.D., Kim Y., Yang M., Vannucci F., Molitor T., Torremorell M., Cheeran M.C.-J. (2020). Immune responses to porcine epidemic diarrhea virus (PEDV) in swine and protection against subsequent infection. PLoS ONE.

[153] Xu X., Du L., Fan B., Sun B., Zhou J., Guo R., Yu Z., Shi D., He K., Li B. (2020). A flagellin-adjuvanted inactivated porcine epidemic diarrhea virus (PEDV) vaccine provides enhanced immune protection against PEDV challenge in piglets. Arch. Virol..

[154] Sato T., Oroku K., Ohshima Y., Furuya Y., Sasakawa C. (2018). Efficacy of genogroup 1 based porcine epidemic diarrhea live vaccine against genogroup 2 field strain in Japan. Virol. J..

[155] Subramaniam S., Yugo D.M., Heffron C.L., Rogers A.J., Sooryanarain H., LeRoith T., Overend C., Cao D., Meng X.J. (2018). Vaccination of sows with a dendritic cell-targeted porcine epidemic diarrhea virus S1 protein-based candidate vaccine reduced viral shedding but exacerbated gross pathological lesions in suckling neonatal piglets. J. Gen. Virol..

[156] Hain K.S., Joshi L.R., Okda F., Nelson J., Singrey A., Lawson S., Martins M., Pillatzki A., Kutish G.F., Nelson E.A. (2016). Immunogenicity of a recombinant parapoxvirus expressing the spike protein of Porcine epidemic diarrhea virus. J. Gen. Virol..

[157] Zhao Y., Fan B., Song X., Gao J., Guo R., Yi C., He Z., Hu H., Jiang J., Zhao L. (2024). PEDV-spike-protein-expressing mRNA vaccine protects piglets against PEDV challenge. mBio.

[158] Chang C.-Y., Hsu W.-T., Chao Y.-C., Chang H.-W. (2018). Display of porcine epidemic diarrhea virus spike protein on baculovirus to improve immunogenicity and protective efficacy. Viruses.

[159] Li Q., Xu Z., Wu T., Peng O., Huang L., Zhang Y., Xue C., Wen Z., Zhou Q., Cao Y. (2018). A flagellin-adjuvanted PED subunit vaccine improved protective efficiency against PEDV variant challenge in pigs. Vaccine.

[160] Pascual-Iglesias A., Sanchez C.M., Penzes Z., Sola I., Enjuanes L., Zuñiga S. (2019). Recombinant Chimeric Transmissible Gastroenteritis Virus (TGEV)—Porcine Epidemic Diarrhea Virus (PEDV) Virus Provides Protection against Virulent PEDV. Viruses.

[161] Decaro N., Campolo M., Mari V., Desario C., Colaianni M.L., Di Trani L., Cordioli P., Buonavoglia C. (2009). A candidate modified-live bovine coronavirus vaccine: Safety and immunogenicity evaluation. New Microbiol..

[162] Koo M., Bendahmane M., Lettieri G.A., Paoletti A.D., Lane T.E., Fitchen J.H., Buchmeier M.J., Beachy R.N. (1999). Protective immunity against murine hepatitis virus (MHV) induced by intranasal or subcutaneous administration of hybrids of tobacco mosaic virus that carries an MHV epitope. Proc. Natl. Acad. Sci. USA.

[163] Lee H.J., Youn H.N., Kwon J.S., Lee Y.J., Kim J.H., Lee J.B., Park S.Y., Choi I.S., Song C.S. (2010). Characterization of a novel live attenuated infectious bronchitis virus vaccine candidate derived from a Korean nephropathogenic strain. Vaccine.

[164] Chandrasekar S.S., Kingstad-Bakke B.A., Wu C.-W., Phanse Y., Osorio J.E., Talaat A.M. (2023). A DNA Prime and MVA Boost Strategy Provides a Robust Immunity against Infectious Bronchitis Virus in Chickens. Vaccines.

[165] Zhai K., Zhang Z., Liu X., Lv J., Zhang L., Li J., Ma Z., Wang Y., Guo H., Zhang Y. (2023). Mucosal immune responses induced by oral administration of recombinant Lactococcus lactis expressing the S1 protein of PDCoV. Virology.

[166] He W., Peng Q., Li J., Huang J., Cai X., Li S., Zhang B., Xiao L., Gao J., Wang C. (2023). Attenuation of a Highly Pathogenic Porcine Deltacoronavirus Strain CZ2020 by a Serial Passage In Vitro. Transbound. Emerg. Dis..

[167] Simões R.S.d.Q., Rodríguez-Lázaro D. (2022). Classical and next-generation vaccine platforms to SARS-CoV-2: Biotechnological strategies and genomic variants. Int. J. Environ. Res. Public. Health.

[168] Rauch S., Jasny E., Schmidt K.E., Petsch B. (2018). New vaccine technologies to combat outbreak situations. Front. Immunol..

[169] Huang Z., Elankumaran S., Yunus A.S., Samal S.K. (2004). A recombinant Newcastle disease virus (NDV) expressing VP2 protein of infectious bursal disease virus (IBDV) protects against NDV and IBDV. J. Virol..

[170] Park M.-S., Steel J., García-Sastre A., Swayne D., Palese P. (2006). Engineered viral vaccine constructs with dual specificity: Avian influenza and Newcastle disease. Proc. Natl. Acad. Sci. USA.

[171] Tang N., Zhang Y., Sadigh Y., Moffat K., Shen Z., Nair V., Yao Y. (2020). Generation of a triple insert live avian herpesvirus vectored vaccine using CRISPR/Cas9-based gene editing. Vaccines.

[172] Kim S.-H., Samal S.K. (2018). Reverse Genetics for Newcastle Disease Virus as a Vaccine Vector. Curr. Protoc. Microbiol..

[173] Khan I., Saeed K., Khan I. (2019). Review nanoparticles: Properties, applications and toxicities. Arab. J. Chem..

[174] Yang Z.-y., Kong W.-p., Huang Y., Roberts A., Murphy B.R., Subbarao K., Nabel G.J. (2004). A DNA vaccine induces SARS coronavirus neutralization and protective immunity in mice. Nature.

[175] Zhang C., Maruggi G., Shan H., Li J. (2019). Advances in mRNA vaccines for infectious diseases. Front. Immunol..

[176] Pardi N., Hogan M.J., Porter F.W., Weissman D. (2018). mRNA vaccines—A new era in vaccinology. Nat. Rev. Drug Discov..

[177] Qin F., Xia F., Chen H., Cui B., Feng Y., Zhang P., Chen J., Luo M. (2021). A guide to nucleic acid vaccines in the prevention and treatment of infectious diseases and cancers: From basic principles to current applications. Front. Cell Dev. Biol..

[178] Xu Q., Ma F., Yang D., Li Q., Yan L., Ou J., Zhang L., Liu Y., Zhan Q., Li R. (2023). Rice-produced classical swine fever virus glycoprotein E2 with herringbone-dimer design to enhance immune responses. Plant Biotechnol. J..

[179] Ma F., Xu Q., Wang A., Yang D., Li Q., Guo J., Zhang L., Ou J., Li R., Yin H. (2024). A universal design of restructured dimer antigens: Development of a superior vaccine against the paramyxovirus in transgenic rice. Proc. Natl. Acad. Sci. USA.

[180] Thakor J.C., Dinesh M., Manikandan R., Bindu S., Sahoo M., Sahoo D., Dhawan M., Pandey M.K., Tiwari R., Emran T.B. (2022). Swine coronaviruses (SCoVs) and their emerging threats to swine population, inter-species transmission, exploring the susceptibility of pigs for SARS-CoV-2 and zoonotic concerns. Vet. Q..

[181] Ma S., Wang M., Zhou J., Feng L. (1994). Adaptation of porcine epidemic diarrhea virus to Vero cells and evaluation of the inactivated vaccine against porcine epidemic diarrhea virus. Chin. Anim. Infect. Dis.

[182] Ma S., Wang M., Feng L., Li W. (1995). Development of bi-combined inactivated vaccine against transmissible gastroenteritis virus and porcine epidemic diarrhea virus. Chin. Anim. Infect. Dis.

[183] Sun R.Q., Cai R.J., Chen Y.Q., Liang P.S., Chen D.K., Song C.X. (2012). Outbreak of porcine epidemic diarrhea in suckling piglets, China. Emerg. Infect. Dis..

[184] Wang D., Fang L., Xiao S. (2016). Porcine epidemic diarrhea in China. Virus Res..

[185] Niu X., Wang Q. (2022). Prevention and Control of Porcine Epidemic Diarrhea: The Development of Recombination-Resistant Live Attenuated Vaccines. Viruses.

[186] Hosseini Z.S., Amani J., Baghbani Arani F., Nazarian S., Motamedi M.J., Shafighian F., Lee S.H., Yang D.-K., Kim H.-H., Cho I.-S. (2018). Efficacy of inactivated variant porcine epidemic diarrhea virus vaccines in growing pigs. Clin. Exp. Vaccine Res..

[187] Opriessnig T., Gerber P.F., Shen H., de Castro A.M.M.G., Zhang J., Chen Q., Halbur P. (2017). Evaluation of the efficacy of a commercial inactivated genogroup 2b-based porcine epidemic diarrhea virus (PEDV) vaccine and experimental live genogroup 1b exposure against 2b challenge. Vet. Res..

[188] Hou Y., Ke H., Kim J., Yoo D., Su Y., Boley P., Chepngeno J., Vlasova A.N., Saif L.J., Wang Q. (2019). Engineering a live attenuated porcine epidemic diarrhea virus vaccine candidate via inactivation of the viral 2’-O-methyltransferase and the endocytosis signal of the spike protein. J. Virol..

[189] Lin C.-M., Ghimire S., Hou Y., Boley P., Langel S.N., Vlasova A.N., Saif L.J., Wang Q. (2019). Pathogenicity and immunogenicity of attenuated porcine epidemic diarrhea virus PC22A strain in conventional weaned pigs. BMC Vet. Res..

[190] Hou Y., Wang Q. (2019). Emerging highly virulent porcine epidemic diarrhea virus: Molecular mechanisms of attenuation and rational design of live attenuated vaccines. Int. J. Mol. Sci..

[191] Langel S.N., Paim F.C., Lager K.M., Vlasova A.N., Saif L.J. (2016). Lactogenic immunity and vaccines for porcine epidemic diarrhea virus (PEDV): Historical and current concepts. Virus Res..

[192] Jang G., Lee D., Lee C. (2022). Development of a Next-Generation Vaccine Platform for Porcine Epidemic Diarrhea Virus Using a Reverse Genetics System. Viruses.

[193] Niu X., Liu M., Yang S., Xu J., Hou Y.J., Liu D., Tang Q., Zhu H., Wang Q. (2023). A recombination-resistant genome for live attenuated and stable PEDV vaccines by engineering the transcriptional regulatory sequences. J. Virol..

[194] Zhang K., Lin S., Li J., Deng S., Zhang J., Wang S. (2022). Modulation of Innate Antiviral Immune Response by Porcine Enteric Coronavirus. Front. Microbiol..

[195] Bijlenga G., Cook J.K., Gelb J., Wit J.D. (2004). Development and use of the H strain of avian infectious bronchitis virus from the Netherlands as a vaccine: A review. Avian Pathol..

[196] Guzmán M., Hidalgo H. (2020). Live Attenuated Infectious Bronchitis Virus Vaccines in Poultry: Modifying Local Viral Populations Dynamics. Animals.

[197] van Beurden S.J., Berends A.J., Krämer-Kühl A., Spekreijse D., Chenard G., Philipp H.-C., Mundt E., Rottier P.J., Verheije M.H. (2018). Recombinant live attenuated avian coronavirus vaccines with deletions in the accessory genes 3ab and/or 5ab protect against infectious bronchitis in chickens. Vaccine.

[198] Jackwood M.W., Clark R., Cheng S., Jordan B.J. (2020). Protection following simultaneous vaccination with three or four different attenuated live vaccine types against infectious bronchitis virus. Avian Pathol..

[199] Gerber J. (1995). Overview of the development of a modified live temperature-sensitive FIP virus vaccine. Feline Pract..

[200] Balint A., Farsang A., Szeredi L., Zadori Z., Belak S. (2014). Recombinant feline coronaviruses as vaccine candidates confer protection in SPF but not in conventional cats. Vet. Microbiol..

[201] Tizard I.R. (2020). Vaccination against coronaviruses in domestic animals. Vaccine.

[202] Addie D.D. (2019). Feline infectious peritonitis: Answers to frequently asked questions concerning FIP and coronavirus. Vet. Nurs. J..

[203] Scott F.W. (1999). Evaluation of risks and benefits associated with vaccination against coronavirus infections in cats. Adv. Vet. Med..

[204] Ithinji D.G., Buchholz D.W., Ezzatpour S., Monreal I.A., Cong Y., Sahler J., Bangar A.S., Imbiakha B., Upadhye V., Liang J. (2022). Multivalent viral particles elicit safe and efficient immunoprotection against Nipah Hendra and Ebola viruses. npj Vaccines.

[205] van Rooij M.H., Schmitz M., Meessen J.M.H., Wouters P.A.W.M., Vrijenhoek M.P., Makoschey B. (2023). Vaccination of calves at day of birth with attenuated vaccines against bovine respiratory syncytial virus, bovine parainfluenza type 3 virus and respiratory bovine coronavirus. Vet. Vaccine.

[206] Pratelli A., Tinelli A., Decaro N., Cirone F., Elia G., Roperto S., Tempesta M., Buonavoglia C. (2003). Efficacy of an inactivated canine coronavirus vaccine in pups. New Microbiol..

[207] Du L., He Y., Zhou Y., Liu S., Zheng B.-J., Jiang S. (2009). The spike protein of SARS-CoV—A target for vaccine and therapeutic development. Nat. Rev. Microbiol..

[208] Promkuntod N., Van Eijndhoven R., De Vrieze G., Gröne A., Verheije M. (2014). Mapping of the receptor-binding domain and amino acids critical for attachment in the spike protein of avian coronavirus infectious bronchitis virus. Virology.

[209] Yao X., Zhu Y., Qiao W.-T., Lu W.-H., Zhang Y.-Q., Li J.-L. (2023). Based on the Results of PEDV Phylogenetic Analysis of the Most Recent Isolates in China, the Occurrence of Further Mutations in the Antigenic Site S1° and COE of the S Protein Which Is the Target Protein of the Vaccine. Transbound. Emerg. Dis..

[210] McBride R., Van Zyl M., Fielding B.C. (2014). The coronavirus nucleocapsid is a multifunctional protein. Viruses.

[211] Sariol A., Perlman S. (2020). Lessons for COVID-19 immunity from other coronavirus infections. Immunity.

[212] Feng W., Xiang Y., Wu L., Chen Z., Li Q., Chen J., Guo Y., Xia D., Chen N., Zhang L. (2022). Nucleocapsid protein of SARS-CoV-2 is a potential target for developing new generation of vaccine. J. Clin. Lab. Anal..

[213] Kim T.W., Lee J.H., Hung C.-F., Peng S., Roden R., Wang M.-C., Viscidi R., Tsai Y.-C., He L., Chen P.-J. (2004). Generation and characterization of DNA vaccines targeting the nucleocapsid protein of severe acute respiratory syndrome coronavirus. J. Virol..

[214] Collisson E.W., Pei J., Dzielawa J., Seo S.H. (2000). Cytotoxic T lymphocytes are critical in the control of infectious bronchitis virus in poultry. Dev. Comp. Immunol..

[215] Seo S.H., Pei J., Briles W.E., Dzielawa J., Collisson E.W. (2000). Adoptive transfer of infectious bronchitis virus primed αβ T cells bearing CD8 antigen protects chicks from acute infection. Virology.

[216] Nakanaga K., Yamanouchi K., Fujiwara K. (1986). Protective effect of monoclonal antibodies on lethal mouse hepatitis virus infection in mice. J. Virol..

[217] Neuman B.W., Kiss G., Kunding A.H., Bhella D., Baksh M.F., Connelly S., Droese B., Klaus J.P., Makino S., Sawicki S.G. (2011). A structural analysis of M protein in coronavirus assembly and morphology. J. Struct. Biol..

[218] Liu J., Sun Y., Qi J., Chu F., Wu H., Gao F., Li T., Yan J., Gao G.F. (2010). The membrane protein of severe acute respiratory syndrome coronavirus acts as a dominant immunogen revealed by a clustering region of novel functionally and structurally defined cytotoxic T-lymphocyte epitopes. J. Infect. Dis..

[219] Pang H., Liu Y., Han X., Xu Y., Jiang F., Wu D., Kong X., Bartlam M., Rao Z. (2004). Protective humoral responses to severe acute respiratory syndrome-associated coronavirus: Implications for the design of an effective protein-based vaccine. J. Gen. Virol..

[220] Dai L., Gao G.F. (2021). Viral targets for vaccines against COVID-19. Nat. Rev. Immunol..

[221] Ruch T.R., Machamer C.E. (2012). The coronavirus E protein: Assembly and beyond. Viruses.

[222] Schoeman D., Fielding B.C. (2019). Coronavirus envelope protein: Current knowledge. Virol. J..

[223] Bhattacharya S., Banerjee A., Ray S. (2021). Development of new vaccine target against SARS-CoV2 using envelope (E) protein: An evolutionary, molecular modeling and docking based study. Int. J. Biol. Macromol..

[224] Zhou H., Fang Y., Xu T., Ni W.J., Shen A.Z., Meng X.M. (2020). Potential therapeutic targets and promising drugs for combating SARS-CoV-2. Br. J. Pharmacol..

[225] Zhu W., Xu M., Chen C.Z., Guo H., Shen M., Hu X., Shinn P., Klumpp-Thomas C., Michael S.G., Zheng W. (2020). Identification of SARS-CoV-2 3CL Protease Inhibitors by a Quantitative High-Throughput Screening. ACS Pharmacol. Transl. Sci..

[226] Mody V., Ho J., Wills S., Mawri A., Lawson L., Ebert M.C.C.J.C., Fortin G.M., Rayalam S., Taval S. (2021). Identification of 3-chymotrypsin like protease (3CLPro) inhibitors as potential anti-SARS-CoV-2 agents. Commun. Biol..

[227] Chakraborty C., Bhattacharya M., Saha A., Alshammari A., Alharbi M., Saikumar G., Pal S., Dhama K., Lee S.-S. (2023). Revealing the structural and molecular interaction landscape of the favipiravir-RTP and SARS-CoV-2 RdRp complex through integrative bioinformatics: Insights for developing potent drugs targeting SARS-CoV-2 and other viruses. J. Infect. Public Health.

[228] Muhammed Y., Yusuf Nadabo A., Pius M., Sani B., Usman J., Anka Garba N., Mohammed Sani J., Opeyemi Olayanju B., Zeal Bala S., Garba Abdullahi M. (2021). SARS-CoV-2 spike protein and RNA dependent RNA polymerase as targets for drug and vaccine development: A review. Biosaf. Health.

[229] Zhu W., Chen C.Z., Gorshkov K., Xu M., Lo D.C., Zheng W. (2020). RNA-Dependent RNA Polymerase as a Target for COVID-19 Drug Discovery. Slas Discov. Adv. Sci. Drug Discov..

[230] Rajpoot S., Alagumuthu M., Baig M.S. (2021). Dual targeting of 3CLpro and PLpro of SARS-CoV-2: A novel structure-based design approach to treat COVID-19. Curr. Res. Struct. Biol..

[231] Mouffouk C., Mouffouk S., Mouffouk S., Hambaba L., Haba H. (2021). Flavonols as potential antiviral drugs targeting SARS-CoV-2 proteases (3CLpro and PLpro), spike protein, RNA-dependent RNA polymerase (RdRp) and angiotensin-converting enzyme II receptor (ACE2). Eur. J. Pharmacol..

[232] Ali Z., Cardoza J.V., Basak S., Narsaria U., Singh V.P., Isaac S.P., França T.C.C., LaPlante S.R., George S.S. (2023). Computational design of candidate multi-epitope vaccine against SARS-CoV-2 targeting structural (S and N) and non-structural (NSP3 and NSP12) proteins. J. Biomol. Struct. Dyn..

[233] Dong Y., Dai T., Wei Y., Zhang L., Zheng M., Zhou F. (2020). A systematic review of SARS-CoV-2 vaccine candidates. Signal Transduct. Target. Ther..

[234] Martinez-Flores D., Zepeda-Cervantes J., Cruz-Resendiz A., Aguirre-Sampieri S., Sampieri A., Vaca L. (2021). SARS-CoV-2 vaccines based on the spike glycoprotein and implications of new viral variants. Front. Immunol..

[235] Ong E., Wong M.U., Huffman A., He Y. (2020). COVID-19 coronavirus vaccine design using reverse vaccinology and machine learning. Front. Immunol..

[236] Singh P.K., Kulsum U., Rufai S.B., Mudliar S.R., Singh S. (2020). Mutations in SARS-CoV-2 leading to antigenic variations in spike protein: A challenge in vaccine development. J. Lab. Physicians.

[237] Singh K., Mehta D., Dumka S., Chauhan A.S., Kumar S. (2023). Quasispecies Nature of RNA Viruses: Lessons from the Past. Vaccines.

[238] Novella I.S., Domingo E., Holland J.J. (1995). Rapid viral quasispecies evolution: Implications for vaccine and drug strategies. Mol. Med. Today.

[239] Andino R., Domingo E. (2015). Viral quasispecies. Virology.

[240] Stevenson-Leggett P., Keep S., Bickerton E. (2020). Treatment with exogenous trypsin expands in vitro cellular tropism of the avian coronavirus infectious bronchitis virus. Viruses.

[241] Donaldson J.M., Kari C., Fragoso R.C., Rodeck U., Williams J.C. (2009). Design and development of masked therapeutic antibodies to limit off-target effects: Application to anti-EGFR antibodies. Cancer Biol. Ther..

[242] Tirado S.M.C., Yoon K.-J. (2003). Antibody-dependent enhancement of virus infection and disease. Viral Immunol..

[243] Yang X., Zhang X., Zhao X., Yuan M., Zhang K., Dai J., Guan X., Qiu H.-J., Li Y. (2022). Antibody-Dependent Enhancement:″Evil ″Antibodies Favorable for Viral Infections. Viruses.

[244] Arvin A.M., Fink K., Schmid M.A., Cathcart A., Spreafico R., Havenar-Daughton C., Lanzavecchia A., Corti D., Virgin H.W. (2020). A perspective on potential antibody-dependent enhancement of SARS-CoV-2. Nature.

[245] Lee W.S., Wheatley A.K., Kent S.J., DeKosky B.J. (2020). Antibody-dependent enhancement and SARS-CoV-2 vaccines and therapies. Nat. Microbiol..

[246] Wan Y., Shang J., Sun S., Tai W., Chen J., Geng Q., He L., Chen Y., Wu J., Shi Z. (2020). Molecular mechanism for antibody-dependent enhancement of coronavirus entry. J. Virol..

[247] Wang S.-F., Tseng S.-P., Yen C.-H., Yang J.-Y., Tsao C.-H., Shen C.-W., Chen K.-H., Liu F.-T., Liu W.-T., Chen Y.-M.A. (2014). Antibody-dependent SARS coronavirus infection is mediated by antibodies against spike proteins. Biochem. Biophys. Res. Commun..

[248] Satoh R., Furukawa T., Kotake M., Takano T., Motokawa K., Gemma T., Watanabe R., Arai S., Hohdatsu T. (2011). Screening and identification of T helper 1 and linear immunodominant antibody-binding epitopes in the spike 2 domain and the nucleocapsid protein of feline infectious peritonitis virus. Vaccine.

[249] Gartlan C., Tipton T., Salguero F.J., Sattentau Q., Gorringe A., Carroll M.W. (2022). Vaccine-associated enhanced disease and pathogenic human coronaviruses. Front. Immunol..

[250] Wang C.-y., Luo Z.-b., Shao G.-q., Hou B. (2022). Genetic and pathogenic characteristics of a novel infectious bronchitis virus strain in genogroup VI (CK/CH/FJ/202005). Vet. Microbiol..

[251] Ren M., Han Z., Zhao Y., Sun J., Liu S., Ma D. (2020). Multiple recombination events between field and vaccine strains resulted in the emergence of a novel infectious bronchitis virus with decreased pathogenicity and altered replication capacity. Poult. Sci..

[252] Zhou P., Li Z., Xie L., An D., Fan Y., Wang X., Li Y., Liu X., Wu J., Li G. (2021). Research progress and challenges to coronavirus vaccine development. J. Med. Virol..

[253] Bashor L., Gagne R.B., Bosco-Lauth A.M., Bowen R.A., Stenglein M., VandeWoude S. (2021). SARS-CoV-2 evolution in animals suggests mechanisms for rapid variant selection. Proc. Natl. Acad. Sci. USA.

[254] Graham R.L., Deming D.J., Deming M.E., Yount B.L., Baric R.S. (2018). Evaluation of a recombination-resistant coronavirus as a broadly applicable, rapidly implementable vaccine platform. Commun. Biol..

[255] Feng C., Shi J., Fan Q., Wang Y., Huang H., Chen F., Tang G., Li Y., Li P., Li J. (2021). Protective humoral and cellular immune responses to SARS-CoV-2 persist up to 1 year after recovery. Nat. Commun..

[256] Stankov M.V., Hoffmann M., Jauregui R.G., Cossmann A., Ramos G.M., Graalmann T., Winter E.J., Friedrichsen M., Ravens I., Ilievska T. (2024). Humoral and cellular immune responses following BNT162b2 XBB. 1.5 vaccination. Lancet Infect. Dis..

[257] Muñoz-Fontela C., Dowling W.E., Funnell S.G., Gsell P.-S., Riveros-Balta A.X., Albrecht R.A., Andersen H., Baric R.S., Carroll M.W., Cavaleri M. (2020). Animal models for COVID-19. Nature.

[258] Li Y.-D., Chi W.-Y., Su J.-H., Ferrall L., Hung C.-F., Wu T.-C. (2020). Coronavirus vaccine development: From SARS and MERS to COVID-19. J. Biomed. Sci..

[259] Keusch G.T., Amuasi J.H., Anderson D.E., Daszak P., Eckerle I., Field H., Koopmans M., Lam S.K., Das Neves C.G., Peiris M. (2022). Pandemic origins and a One Health approach to preparedness and prevention: Solutions based on SARS-CoV-2 and other RNA viruses. Proc. Natl. Acad. Sci. USA.

[260] Ledesma-Feliciano C., Chapman R., Hooper J.W., Elma K., Zehrung D., Brennan M.B., Spiegel E.K. (2023). Improved DNA vaccine delivery with needle-free injection systems. Vaccines.

[261] Lei Z., Zhu L., Pan P., Ruan Z., Gu Y., Xia X., Wang S., Ge W., Yao Y., Luo F. (2023). A vaccine delivery system promotes strong immune responses against SARS-CoV-2 variants. J. Med. Virol..

